# LXR/RXR signaling and neutrophil phenotype following myocardial infarction classify sex differences in remodeling

**DOI:** 10.1007/s00395-018-0699-5

**Published:** 2018-08-21

**Authors:** Kristine Y. DeLeon-Pennell, Alan J. Mouton, Osasere K. Ero, Yonggang Ma, Rugmani Padmanabhan Iyer, Elizabeth R. Flynn, Ingrid Espinoza, Solomon K. Musani, Ramachandran S. Vasan, Michael E. Hall, Ervin R. Fox, Merry L. Lindsey

**Affiliations:** 1 Mississippi Center for Heart Research, Department of Physiology and Biophysics, University of Mississippi Medical Center, 2500 North State St., Jackson, MS 39216-4505 USA; 2 Research Service, G.V. (Sonny) Montgomery Veterans Affairs Medical Center, Jackson, MS USA; 3 Department of Preventive Medicine and Cancer Institute, UMMC, Jackson, MS USA; 4 Jackson Heart Study, UMMC, Jackson, MS USA; 5 Section of Preventive Medicine and Epidemiology and Cardiology, Department of Medicine, Boston University School of Medicine, Boston, USA; 6 Division of Cardiology, UMMC, Jackson, MS USA

**Keywords:** Mice, Humans, Sex differences, Neutrophils, LXR/RXR, Proteomics, Big data

## Abstract

Sex differences in heart failure development following myocardial infarction (MI) are not fully understood. We hypothesized that differential MI signaling could explain variations in outcomes. Analysis of the mouse heart attack research tool 1.0 (422 mice; young = 5.4 ± 0.1; old = 23.3 ± 0.1 months of age) was used to dissect MI signaling pathways, which was validated in a new cohort of mice (4.8 ± 0.2 months of age); and substantiated in humans. Plasma collected at visit 2 from the MI subset of the Jackson Heart Study (JHS; a community-based study consisting of middle aged and older adults of African ancestry) underwent glycoproteomics grouped by outcome: (1) heart failure hospitalization after visit 2 (*n* = 3 men/12 women) and (2) without hospitalization through 2012 (*n* = 24 men/21 women). Compared to young male mice, the infarct region of young females had fewer, but more efficient tissue clearing neutrophils with reduced pro-inflammatory gene expression. Apolipoprotein (Apo) F, which acts upstream of the liver X receptors/retinoid X receptor (LXR/RXR) pathway, was elevated in the day 7 infarcts of old mice compared to young controls and was increased in both men and women with heart failure. In vitro, Apo F stimulated CD36 and peroxisome proliferator-activated receptor (PPAR)γ activation in male neutrophils to turn off NF-κB activation and stimulate LXR/RXR signaling to initiate resolution. Female neutrophils were desensitized to Apo F and instead relied on thrombospondin-1 stimulation of CD36 to upregulate AMP-activated protein kinase, resulting in an overall better wound healing strategy. With age, female mice were desensitized to LXR/RXR signaling, resulting in enhanced interleukin-6 activation, a finding replicated in the JHS community cohort. This is the first report to uncover sex differences in post-MI neutrophil signaling that yielded better outcomes in young females and worse outcomes with age.

**Electronic supplementary material:**

The online version of this article (10.1007/s00395-018-0699-5) contains supplementary material, which is available to authorized users.

## Introduction

The average age for a first myocardial infarction (MI) is 65 years for men and 72 years for women [4]. Differences between men and women in long-term prognosis after MI have been attributed to age, clinical presentation, and treatment utilization [7, 60]. Following MI, the left ventricle (LV) undergoes a wound healing response that involves necrotic tissue removal and deposition of a replacement scar composed of extracellular matrix proteins. Immune cells polarize to a pro-inflammatory state and release enzymes such as matrix metalloproteinases (MMPs) and reactive oxygen species (ROS) that catalyze necrotic tissue removal [16, 18, 48]. Infiltrating leukocytes also secrete cytokines and growth factors such as pro-inflammatory interleukin (IL)1β and reparative transforming growth factor β [18, 81].

Women have a more temperate response to inflammatory stimuli; for example, in sepsis and atherosclerosis, they have lower pro-inflammatory leukocyte-mediated inflammation and accelerated resolution of inflammation compared to men [66, 71]. Whether and to what extent sex differences exist in the post-MI inflammatory and wound healing response has not been previously examined.

The goal of this study, accordingly, was to assess sex differences in post-MI responses using a combination of experimental MI in mice and human post-MI proteomic data from a well-characterized longitudinal cardiovascular study of an African American population: the Jackson Heart Study. We hypothesized that differential signaling activation following MI could explain sex differences in cardiac remodeling in mice and humans. We used convergent methods to compare alterations in LV gene with plasma and LV protein and a cross-translational approach to compare results in mice and humans, and performed cell phenotyping and physiology analyses to evaluate common and contrasting signaling responses.

## Materials and methods (detailed methods in supplemental materials)

### Analysis of the mouse heart attack research tool (mHART) 1.0 database [17]

**Fig. 1:**
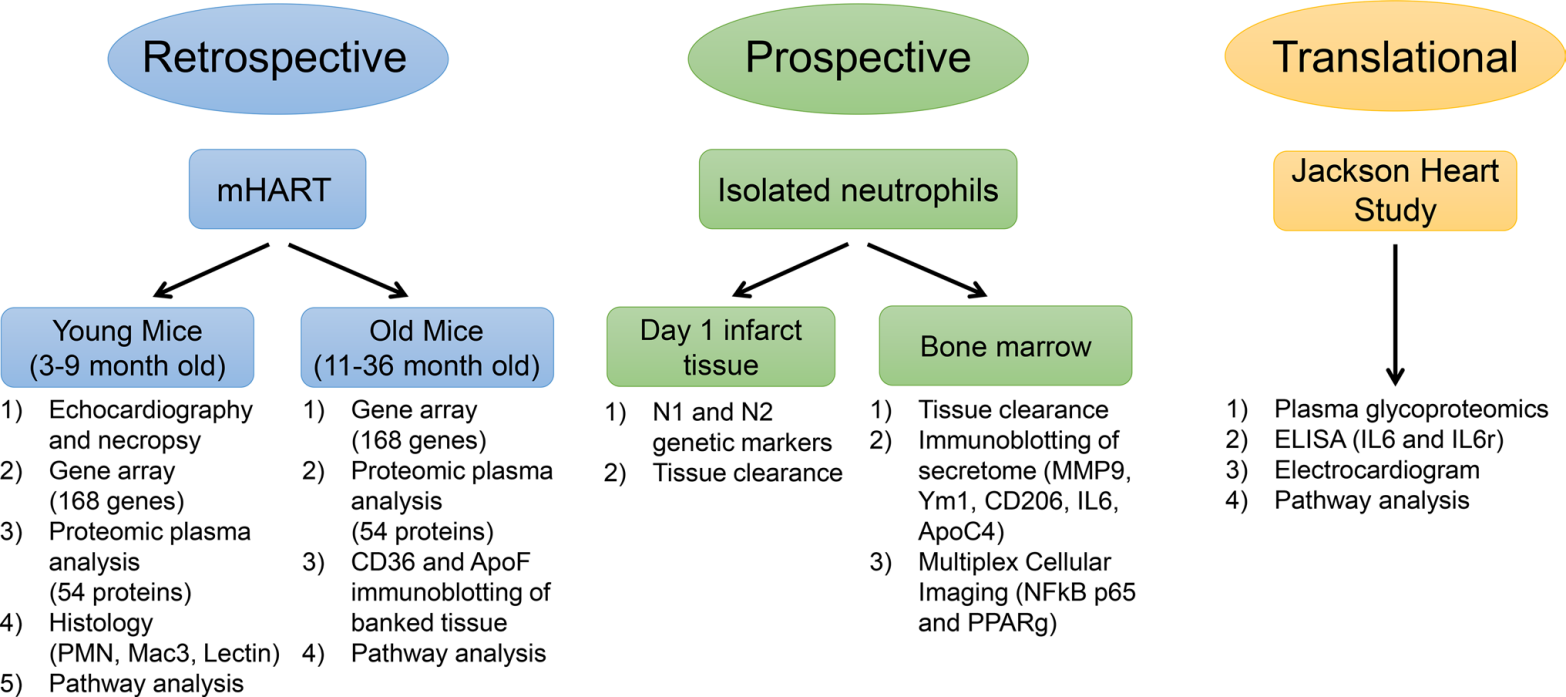
Experimental design. Retrospective (mHART), prospective (isolated neutrophils), and translational (Jackson Heart Study) data were collected and analyzed to dissect the mechanism of sex differences in MI remodeling leading to heart failure

The mHART database consists of data collected from projects published since 2007 [17]. The article describing the development of the database included a preliminary example of dilation responses. Here, we expand the original observation to identify molecular and cellular mechanisms that explain sex differences in MI outcomes. Data were collected for inclusion in the database from projects published in our lab for male and female mice ranging from 3 to 36 months of age [10, 13, 14, 31, 32, 50, 52, 53, 81, 86–88]. For this study, we analyzed data from 422 male and female C56BL/6 J wild-type (WT) mice (Online Table 1 and Fig. 1). At autopsy, cardiac rupture in non-surviving mice was confirmed by the presence of coagulated blood in the thoracic cavity or observation of the rupture site on the LV.

#### Sample selection

The database was accessed on April 15, 2017. Of 2095 mice downloaded, 759 were WT post-MI mice. Of 759 mice, 422 had echocardiography and plasma or gene RT-PCR data at days 0 (*n* = 161; 70 F and 91 M), 1 (*n* = 38; 15 F and 23 M), 3 (*n* = 46; 18 F and 28 M), and 7 (*n *= 95; 47 F and 48 M) post-MI. Both MI surgeries and echocardiography were performed according to established guidelines [49, 51].

#### Immunoblotting of banked tissue

Immunoblotting was performed to evaluate Apo F and CD36 in the LV infarct of young and old male and female hearts (*n *= 5/sex/day post-MI; Online Fig. 1a).

### Neutrophil cell isolation

To explore new hypotheses generated by the database analysis, WT mice (3–6 months-old) were used in accordance with the Guide for the Care and Use of Laboratory Animals (Eighth edition) in a protocol approved by our IACUC. Mice were subjected to permanent occlusion of the left anterior descending coronary artery [32].

#### Neutrophil phenotyping

Neutrophils were isolated from the infarct at day 1 post-MI (*n* ≥ 8 mice/group) [54]. RNA array experiments were performed according to the Minimum Information for Publication of Quantitative Real-Time PCR Experiments (MIQE) guidelines with one exception; hypoxanthine guanine phosphoribosyl transferase 1 (*Hprt1*) was the only reference gene used, as *GusB*, *Hsp90ab1*, *Actb*, and *Gapdh* have all been shown to change after MI [32]. Immunoblotting results for isolated neutrophils are shown in Online Fig. 1b.

#### In vitro neutrophil stimulation

To establish causality, neutrophils were isolated from the bone marrow of no MI naive mice [54]. Cells (5 × 10^5^ condition) were stimulated with Apo F with or without CD36 blocking antibody (CD36i) for 15 min at 37 °C.

#### Tissue clearance capacity

Ex vivo neutrophils and macrophages isolated from the day 1 LV infarct were plated in duplicate (1.0 × 10^5^ cells/well) onto gelatin coated 48-well plates. In vitro neutrophils were stimulated with Apo F or 50 µL of the secretome (10% final volume). Tissue clearance was calculated by normalizing absorbance of the negative controls (wells without cells) using the following formula: 1-[(sample Abs/(negative control Abs)].

#### Multiplex cellular imaging

Opal multiplex immunohistochemistry was performed on isolated neutrophils, using antibodies specific for nuclear factor (NF)κB-p65 or peroxisome proliferator-activated receptor (PPAR)γ, with DAPI as a nuclear counterstain and according to established guidelines for antibody use [5]. Images were acquired on the Mantra™ Quantitative Pathology Imaging microscope. The number of cells with nuclear staining were counted and calculated as percentage of total cell numbers.

### Jackson Heart Study data

The Jackson Heart Study is a community-based cohort study that consists of 5306 African American participants between the ages of 21 and 94 years [83]. Incident heart failure events were formally adjudicated by Jackson Heart Study from January 1, 2005 to December 31, 2012. Participants who did not have an MI before visit 2, had heart failure at or before visit 2, were diagnosed with MI after 2008, or did not have plasma collected at visit 2 were excluded, leaving a final sample size of 60 participants, which were analyzed using glycoproteomics as described previously [15, 19, 76, 77]. The mass spectrometry proteomics data have been deposited to the ProteomeXchange Consortium via the PRIDE [80] partner repository with the dataset identifier PXD009870.

### Statistics

Animal and human datasets underwent functional analysis using Ingenuity Pathway Analysis (IPA; QIAGEN Redwood City; www.qiagen.com/ingenuity). Multiple-testing corrected p values were calculated in IPA using the Benjamini–Hochberg method. Fold change was calculated for each sex by normalizing MI + HF data to the respective MI only samples for the clinical samples. Mouse data were normalized to their respective day 0 dataset. This allowed us to focus on changes that predict heart failure and LV remodeling. The most statistically significant canonical pathways identified are ranked according to p value. The ratio given is the number of pathway proteins identified in the glycoproteomics dataset divided by the total number of proteins in that pathway, which reflects percentage of pathway coverage. The Z score was calculated based on expected relationship direction and observed gene expression differences in MI vs MI + HF for each sex.

Causal and upstream network analyses were employed to identify molecules upstream of the measured genes and proteins in the dataset that explained the observed expression changes. IPA statistical analysis scored regulators whose network connections were highly unlikely to occur in a random model. A cut off of the overlap p value measuring enrichment of network-regulated genes and the activation *Z* score was used to identify regulating molecules and to predict regulator activation state. Heat maps and volcano plots were constructed using a statistical program available in the Metaboanalyst 3.0 package (www.metaboanalyst.ca/) [82, 85].

All statistical analyses were performed by investigators blinded to groups. Animal studies are presented as mean ± SEM and community-based cohort data as mean ± SD. Two group comparisons were analyzed by Student’s *t* test. Multiple group comparisons were analyzed using one-way ANOVA, followed by the Student–Newman-Keuls when the Bartlett’s variation test was passed, or the nonparametric Kruskal–Wallis test, followed by Dunn post hoc test when the Bartlett’s variation test did not pass. Statistical significance was set at p < 0.05.

### Study approval

This study complies with the Declaration of Helsinki [84]. All animal procedures were approved by the Institutional Animal Care and Use Committee at the University of Mississippi Medical Center in accordance with the Guide for the Care and Use of Laboratory Animals and followed the ARRIVE guidelines [37]. The Jackson Heart Study was approved by the institutional review boards of the University of Mississippi Medical Center, Jackson State University, and Tougaloo College. All participants provided written informed consent. This study was approved as IRB #2014-0274.

## Results

### Post-MI outcome was improved and inflammation attenuated in young female mice

**Fig. 2:**
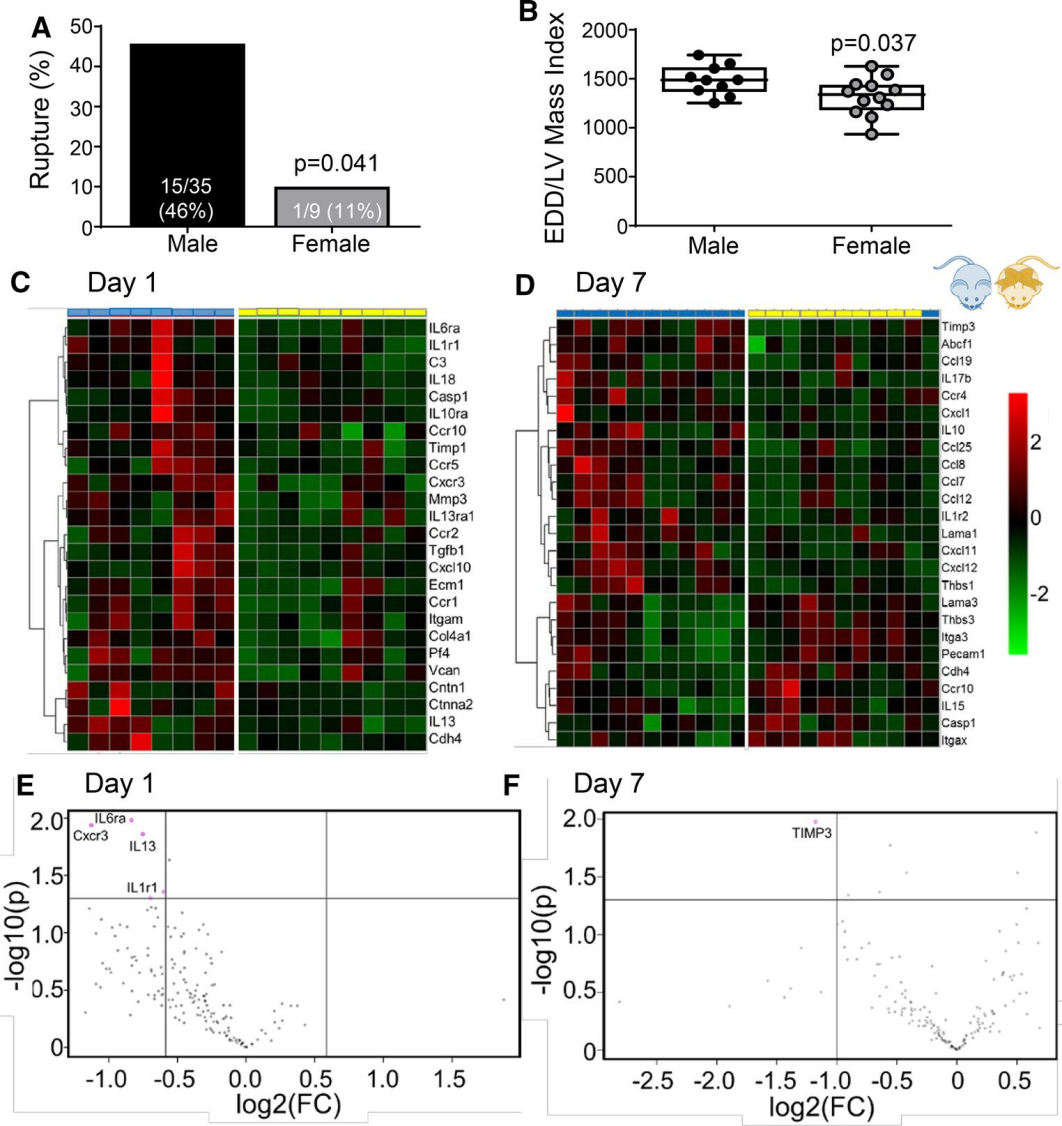
Post-myocardial infarction (MI) inflammation was attenuated in young female mice. **a** Cardiac rupture and **b** end diastolic dimension (EDD) was lower in female mice, *n* ≥ 10/sex. **c**, **d** Bioinformatic analysis of 165 inflammatory and extracellular matrix genes (RT-PCR results from the mHART database) indicated accelerated post-MI inflammation resolution in female mice, *n* ≥ 8/sex. The top 25 genes based on p value were clustered using Euclidean distance measurements and average linkage. **e** At day 1 post-MI, infarct gene levels of interleukin (IL)6ra, Cxcr3, IL13, and IL1r1 were > twofold lower in female mice. **f** At day 7 post-MI, tissue inhibitor of matrix metalloproteinase-3 gene expression was > twofold lower. Rupture rates were analyzed by Fisher’s exact test and multiple group comparisons by one-way ANOVA and Student–Newman–Keuls post-test. Heat maps and volcano plots were constructed using a visualization program available in the Metaboanalyst 3.0 package (www.metaboanalyst.ca/); *p < 0.05 vs. respective day 0; ^#^*p* < 0.05 vs male mice

At day 7 post-MI, young female mice showed improved survival (74 vs 40% for males; *p* = 0.003), less rupture (11 vs. 46% for males, *p* = 0.041) and attenuated LV dilation, even when normalized to LV mass index (Fig. 2a, b). Infarct areas were similar between sexes (Online Table 2), indicating observed changes were not due to differences in initial injury stimulus.

**Fig. 3:**
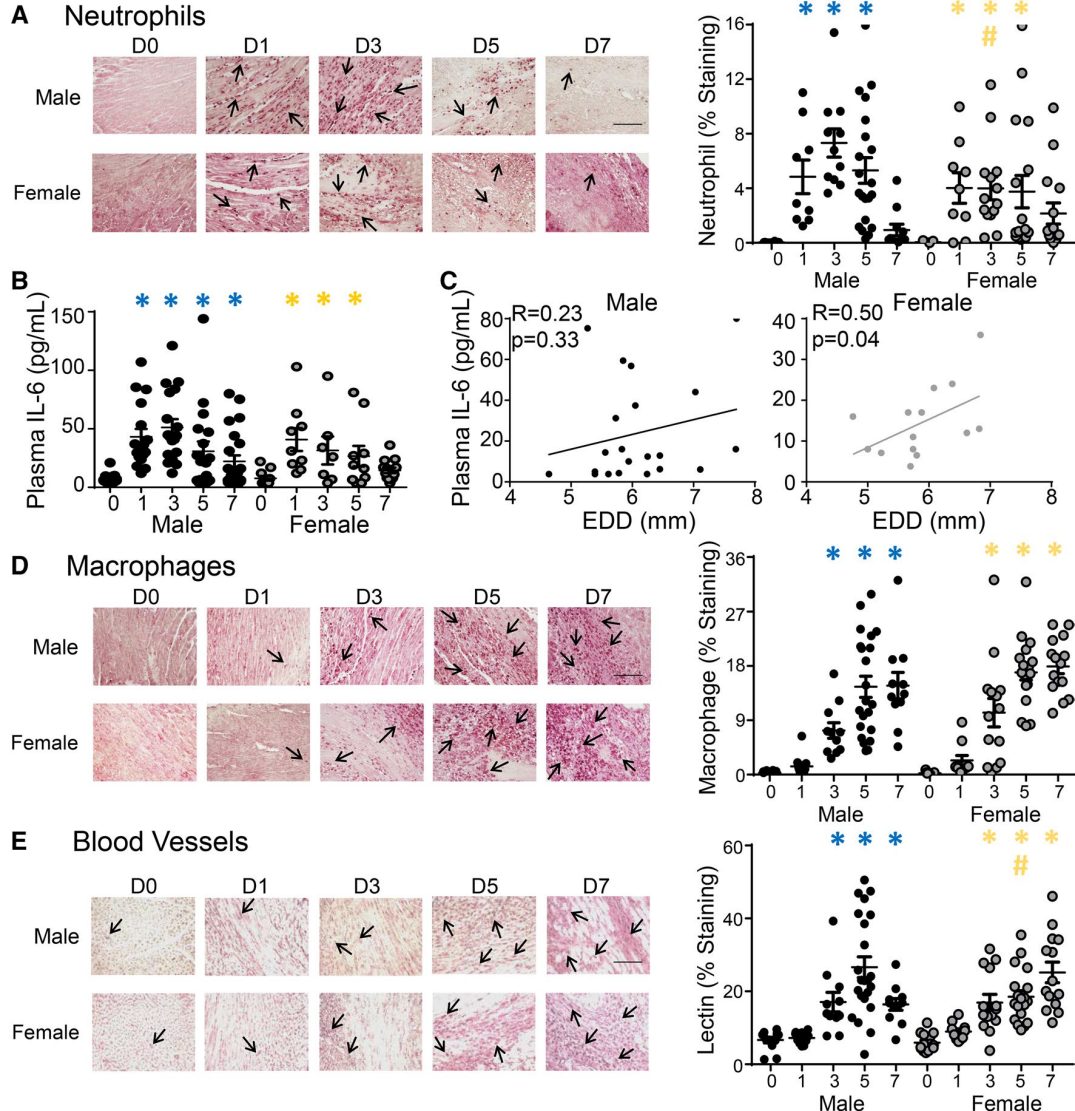
Sex differences in neutrophil recruitment and vessel formation in the infarct. **a** Neutrophil numbers in the day 3 infarct were lower in female compared to male mice, *n* ≥ 10/sex/day (immunohistochemistry results from the mHART database). **b** Plasma interleukin (IL)6 concentrations (plasma proteomic profiling from the mHART database) increased at day 1 after myocardial infarction (MI) and peaked at day 3. In male mice, IL6 remained elevated at day 7 post-MI whereas, in female mice, IL6 had returned to baseline levels by day 7 post-MI, *n* ≥ 8/sex/day. **c** Plasma IL6 correlated with end diastolic dimension (EDD) in female but not male mice, indicating IL6 was a critical regulation node of cardiac remodeling in females, *n* ≥ 14/sex. **d** No difference was observed in macrophage numbers, *n* ≥ 10/sex/day. **e** Lectin staining showed slower acceleration of vessel formation in female mice, *n* ≥ 10/sex/day. Data are shown as mean ± SEM; Scale bar = 60 um; Multiple group comparisons were analyzed by one-way ANOVA and Student–Newman–Keuls post-test. *p < 0.05 vs. respective day 0; ^#^*p* < 0.05 vs male mice at respective post-MI day

Inflammation was robustly upregulated in males compared to no MI controls, and this induction was blunted in female mice (Fig. 2c–f). The main drivers of sex-dependent LV remodeling regulation at day 1 were IL6Rα, Cxcr3, IL13, and IL1r1 (Fig. 2e; all > twofold lower and p < 0.05 in females compared to males). In addition to these 4 genes, Cxcl4 and tissue inhibitor of matrix metalloproteinase (TIMP)-3 were lower in young female mice compared to males (both *p* < 0.05). At day 7, TIMP-3, ATP binding cassette subfamily F member-1, Ccl25, Cxcl12, integrin-a3, and thrombospondin (TSP)-1 and -3 were lower in female mice compared to male mice (Fig. 2f, all *p* < 0.05). Post-MI neutrophil infiltration was robust by day 1 and returned to baseline levels by day 7 in both male and female young mice (Fig. 3a and Online Fig. 2). While the time course was similar, males had higher neutrophil numbers at day 3 compared to females, consistent with the higher pro-inflammatory status and previous studies showing more cardiac rupture and neutrophil infiltration in male mice [9].

While post-MI plasma IL6 concentrations increased at day 1 and peaked at day 3 in both sexes (Fig. 3b), females had reduced IL6 receptor (IL6R) gene and protein expression in the infarct at day 1 compared to males (*p* < 0.05; Fig. 2e and Online Fig. 1a). IL6R gene expression at day 3 post-MI correlated with neutrophil numbers in female mice (*r* = 0.85; *p* = 0.03) but not male mice (*r* = 0.67; *p *= 0.33), consistent with lower neutrophil numbers in females in response to IL6 chemoattraction [22, 40, 70]. In young female mice, plasma IL6 protein returned to baseline by day 7 post-MI; whereas IL6 resolution was delayed and remained elevated at day 7 in males. Female mice were more sensitive to sustained IL6, as increased plasma IL6 at day 7 correlated with increased end diastolic dimension in females, but not males (Fig. 3c). Macrophage (Mac-3 +) cell numbers were similar in male and female mice, both for timing and magnitude of response (Fig. 3d). Vessel formation in the infarct zone, denoted by Griffonia (Bandeiraea) Simplicifolia Lectin I (GSL)-lectin + cells, occurred earlier in male mice, as vessel numbers peaked at day 5 and were higher compared to female mice (Fig. 3e).

**Fig. 4:**
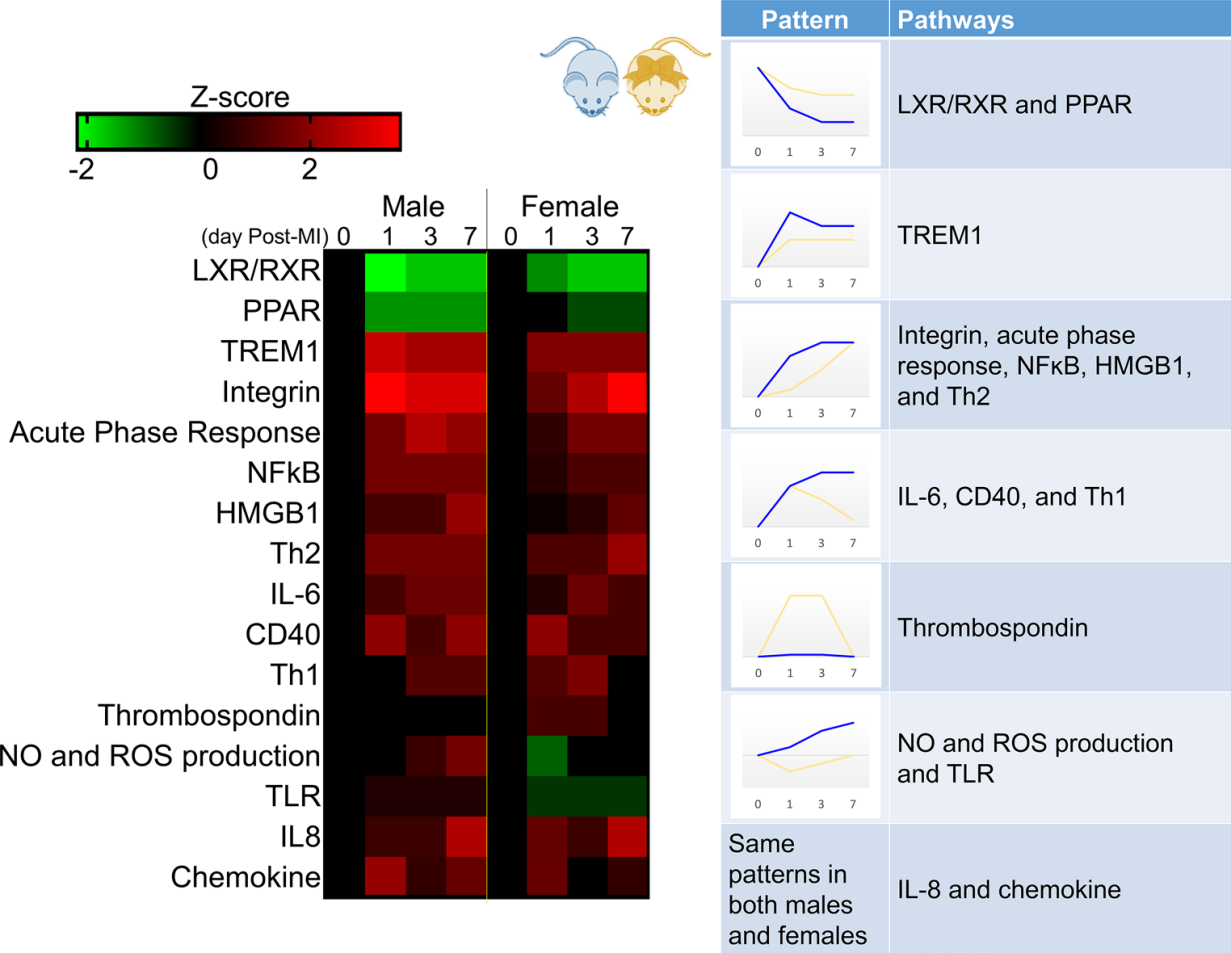
Pathway activation after myocardial infarction (MI) revealed sex divergence. By time course evaluation of 165 inflammatory and extracellular matrix genes and 56 plasma analytes (from the mHART database), male mice (blue) had a rapid and robust inflammatory response to MI, whereas female mice (yellow) showed a more gradual induction. IL-8 and chemokine pathways were similar in direction of change and magnitude between young males and females. Liver X receptors/retinoid X receptor (LXR/RXR), peroxisome proliferator-activated receptor (PPAR), and triggering receptor expressed on myeloid cells (TREM)1 showed a similar timeline, but lower magnitude of response in females. The integrin acute phase response, nuclear factor (NF)-kB, high-mobility group protein 1 (HMGB1), and Th2 pathways increased slower in females compared to males. Interleukin (IL)6, CD40, and Th1 pathways increased quickly, peaking at day 1 before returning to baseline levels in females while remaining elevated in males. TSP signaling was the only pathway elevated solely in females. Nitric oxide (NO) and reactive oxygen species (ROS) production and toll-like receptor (TLR) activation decreased in females and increased in males. *n* ≥ 15/sex/day; Multiple-testing corrected *p* values were calculated using the Benjamini–Hochberg method, and the Z score was calculated in IPA. Data were normalized to respective day 0 for each sex to calculate fold change in response to MI

Canonical pathway analysis of 165 inflammatory and extracellular matrix genes and 56 plasma proteins measured over the day 0–7 post-MI time course identified 16 pathways associated with LV remodeling in both males and females (Fig. 4). Two pathways (IL-8 and chemokine) were similar in direction of change and magnitude between young males and females, designating shared common regulation. The other 14 pathways followed one of the five distinct patterns: (1) same direction with lower magnitude of change in females (liver X receptors/retinoid X receptor (LXR/RXR), peroxisome proliferator-activated receptor (PPAR), and triggering receptor expressed on myeloid cells (TREM)-1); (2) same direction with slower increase in females (integrin, acute phase response, NF-ĸB, high-mobility group protein 1, Th2); (3) same direction with earlier resolution in females (IL6, CD40, Th1); (4) increase in females only (TSP); and (5) opposite direction of magnitude [nitric oxide (NO) and reactive oxygen species (ROS) production and toll-like receptor (TLR) activation]. Combined, these results indicate males had a more robust response to ischemia induced cardiac injury resulting in poor survival and adverse remodeling compared to females.

### Post-MI sex differences were dependent on neutrophil signaling

**Fig. 5:**
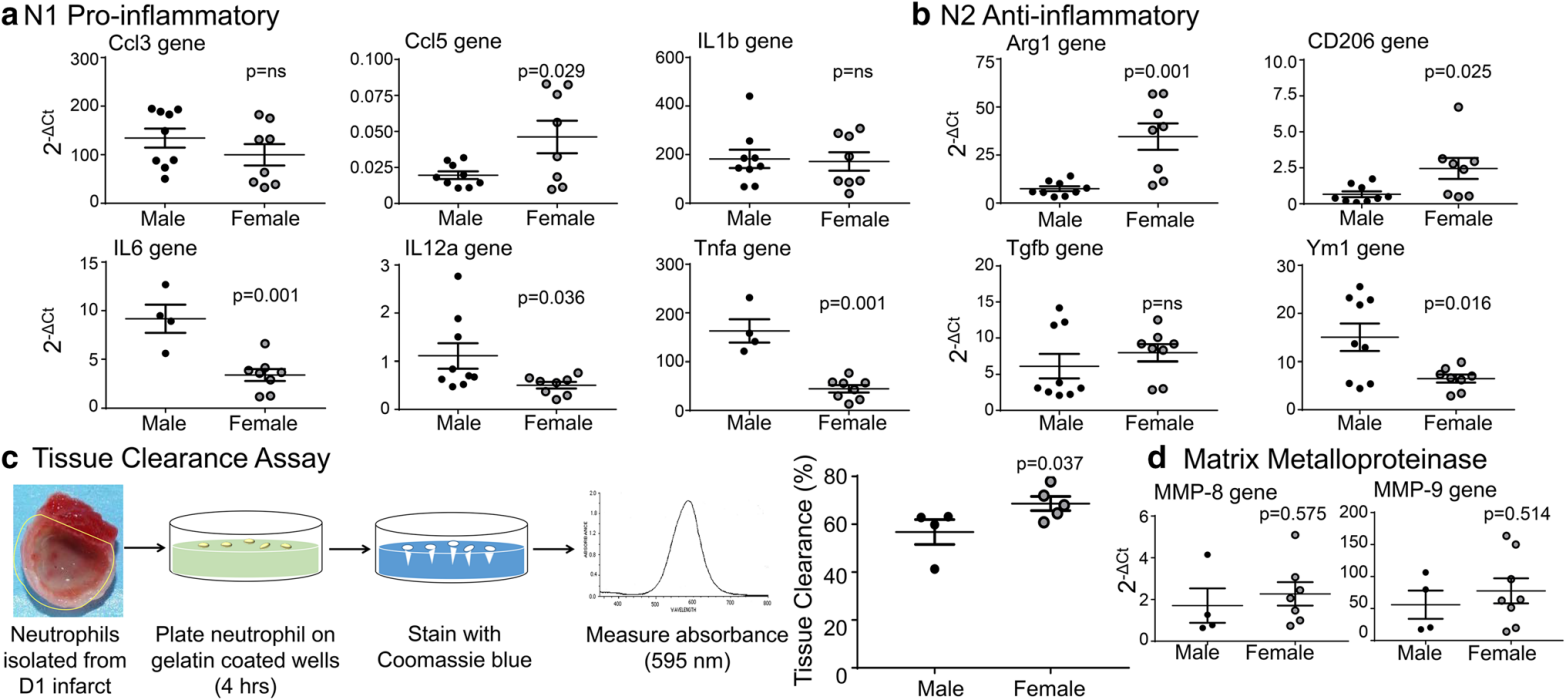
Female neutrophils were more efficient at clearing necrotic tissue. **a** Females had lower pro-inflammatory N1 gene markers and **b** increased anti-inflammatory N2 gene markers in neutrophils isolated from day 1 post-MI left ventricle compared to males; n ≥ 4/sex. **c** Day 1 post-MI neutrophils from female mice cleared necrotic debris (by tissue clearance assay) more efficiently than neutrophils from male mice, facilitating inflammation resolution, n ≥ 4/sex. **d** No change was observed in matrix metalloproteinase (MMP)-8 or –9 gene expression, *n* ≥ 4/sex. For two group comparisons, the nonparametric Wilcoxon rank sum test was used. *NS* not significant

Previously, we showed that neutrophils can polarize to a pro-inflammatory (N1) or anti-inflammatory (N2) phenotype [54]. Out of six N1 markers, day 1 neutrophils from females had elevated Ccl5 gene and reduced Ccl5 protein expression compared to neutrophils isolated from the infarct of males (Fig. 5a). The divergence in gene and protein expression indicates that females may have a feedback loop to signal induction of gene expression due to reduced protein. IL6, IL12a, and Tnfα gene and protein were lower per cell in neutrophils isolated from female mice at day 1 compared to male neutrophils (Fig. 5b; *p *< 0.05 and Online Fig. 1b). IL1β was not different between sexes. Out of the four N2 gene markers assayed, Arg1 and CD206 were higher, Ym1 was lower, and Tgfβ was not different in day 1 female neutrophils (Fig. 5c). At the protein level, only CD206 was elevated in neutrophils isolated at day 1 post-MI from female mice. These results indicate neutrophils from females show reduced N1 and greater N2 polarization.

By tissue clearance assay, neutrophils from the infarct of females showed greater tissue clearance capacity per cell compared to neutrophils isolated from males (Fig. 5d) When we assessed the tissue clearance capacity of macrophages isolated from the day 1 infarcts, no significant difference was found between sexes (*p *= 0.52). To assess if neutrophil mediated-tissue clearance was due to elevated MMPs, MMP-8 and -9 gene expression was evaluated in day 1 neutrophils (Fig. 5e). Neither MMP-8 nor -9 were different between sexes, indicating female neutrophils are more efficient at removal of necrotic tissue without inducing excessive extracellular matrix degradation. Overall, post-MI female neutrophils displayed lower inflammatory markers, which may result in less overall damage to the myocardium.

### With aging, female mice lose the ability to activate LXR/RXR signaling

**Fig. 6:**
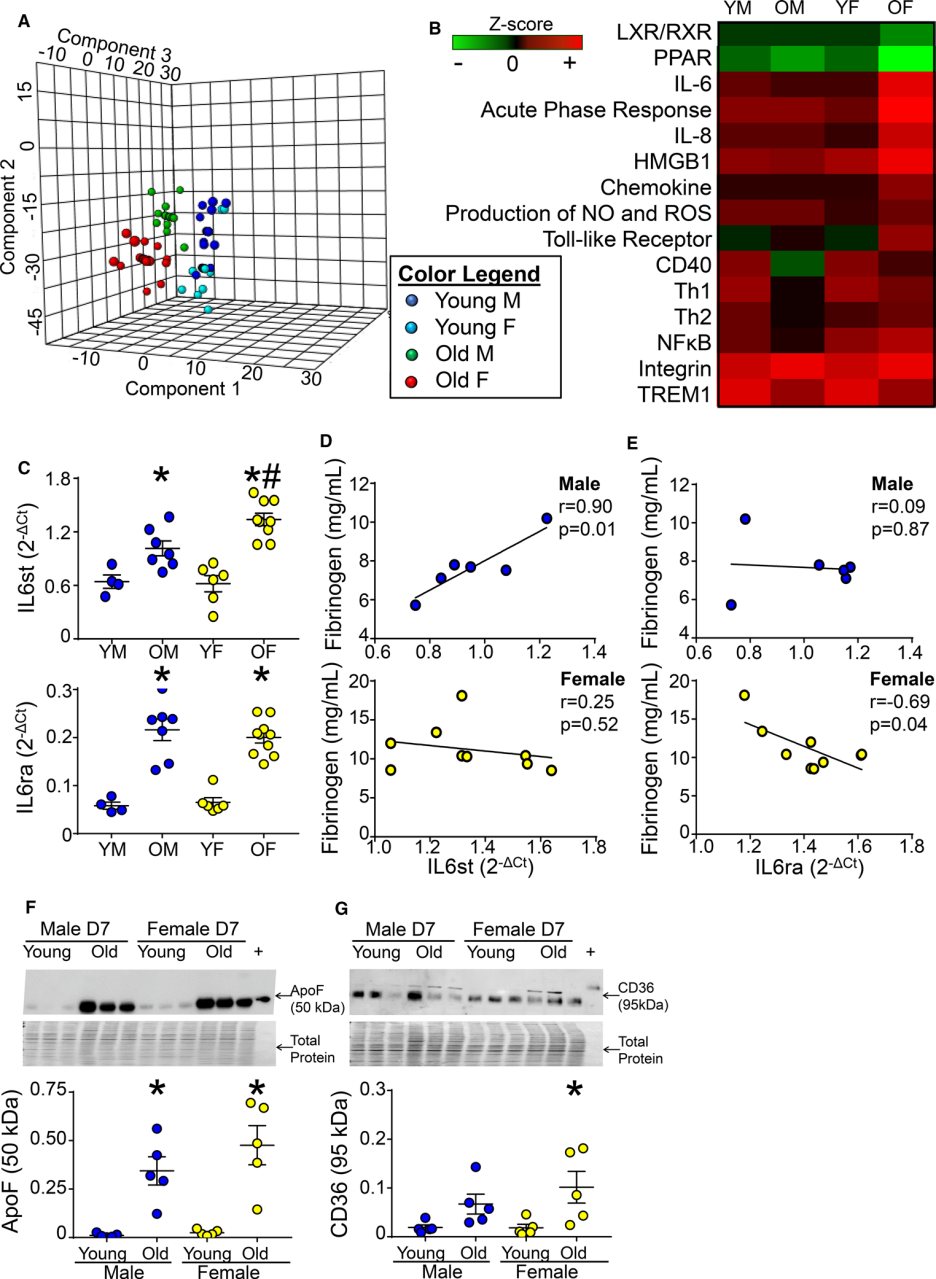
With age, female mice lost the ability to activate LXR/RXR signaling. **a** Partial least squares discriminant analysis of 165 inflammatory and extracellular matrix genes and 56 plasma analytes (from the mHART database) from young (3–9 month-old) and old (11–36 month-old) mice revealed day 7 post-MI young females clustered with day 7 post-MI young males, whereas day 7 post-MI old females and males clustered in two distinct groups, *n* ≥ 18/sex/day/age group. **b** With age LXR/RXR and PPAR signaling activation decreased and interleukin (IL)6, the acute phase response, IL8 signaling, high-mobility group protein 1 (HMGB1), chemokine, and production of NO and ROS pathways increased in females only, *n* ≥ 18/sex/day/age. *Z* score was calculated in IPA. Data were normalized to respective day 0 for each sex and age group. **c** IL6st and IL6ra mRNA at day 7 post-MI increased with age in both sexes. IL6st expression was exacerbated in old females compared to old males, *n* ≥ 4/sex/day/age. **d** IL6st mRNA correlated with plasma levels of fibrinogen, an LXR/RXR pathway protein, in old male mice, but not females whereas, **e** IL6ra mRNA negatively correlated with fibrinogen in old female mice but not old male mice; *n* ≥ 10/sex. Immunoblotting of **f** Apo F and **g** CD36 indicated increased levels in day 7 LV infarct of old female mice compared to young mice; n = 5/group; Multiple group comparisons were analyzed by one-way ANOVA with Student–Newman–Keuls post-test. *p < 0.05 vs. young mice of respective sex; ^#^*p* < 0.05 vs old male mice

Women show greater protection from cardiovascular disease until menopause. Mice reach the endocrine equivalent of human perimenopause by 9 months of age [23, 26, 56]. To dissect post-MI age vs. hormonal effects, we compared young (3–9 month-old) and old (11–36 month-old) mice within the mHART 1.0 database. Sparse partial least squares discriminant analysis revealed day 7 post-MI young females clustered with day 7 post-MI young males, whereas day 7 post-MI old females and males clustered as two distinct groups (Fig. 6a) signifying male and female differences became more prominent with age.

With age, activation of a number of pathways were amplified in females compared to their young female counterparts: IL6 (*Z* score = 1.0 for young, 3.5 for old), acute phase response (*Z* score = 1.6 for young, 3.9 for old), IL8 (*Z* score = 0.8 for young, 3.1 for old), high-mobility group protein 1 (*Z* score = 2.5 for young, 3.7 for old), chemokine (*Z* score = 0.7 for young, 2.1 for old), and production of NOS and ROS (Z score = 0.8 for young, 1.6 for old; Fig. 6b). These results revealed a more robust response to ischemia in females with age. Compared to young females, two pathways downregulated in old females were LXR/RXR (*Z* score = − 0.7 for young, − 1.7 for old females) and PPAR (*Z* score = − 1.3 for young, − 3.2 for old females), signifying age-related desensitization. Male mice had no change in any of these pathways with age. Both old male and female mice had exacerbated toll-like receptor and integrin signaling and blunted CD40, TREM, and Th1 signaling responses to MI compared to their young counterparts. In contrast, MI activation of Th2 and NF-κB signaling were amplified in females and blunted in males with age.

IL6 is a hallmark trigger for the acute-phase response and is inhibited by LXR/RXR activation [3, 41]. To determine if age-induced deactivation of LXR/RXR signaling in females explained the increase in IL6 signaling, infarct expression of IL6 signal transduction (IL6st) and IL6 receptor alpha (IL6ra) genes at day 7 post-MI were evaluated. MI-induced increases in IL6st and IL6ra were exacerbated with age in females, and IL6st was elevated in old females compared to old males (Fig. 6c). IL6st correlated with circulating fibrinogen, a marker for LXR/RXR activation, in old male but not old female mice (Fig. 6d), whereas IL6ra negatively correlated with fibrinogen in old female mice but not old males (Fig. 6e).

Apolipoprotein (Apo) F (lipid transfer inhibitory protein) regulates high-density lipoprotein metabolism and is an upstream activator of LXR/RXR signaling [45]. With age, apo F was similarly elevated in the day 7 infarcts of both sexes compared to young controls (Fig. 6f), indicating sex differences in signaling occur downstream of apo F. CD36 acts downstream of apo F and is known to activate LXR/RXR. Old females had higher CD36 surface expression compared to young females (Fig. 6g) most likely as a compensation mechanism resulting from decreased LXR/RXR signaling with age.

### Apo F stimulation of young male neutrophils activated LXR/RXR signaling through CD36 and PPARγ activation to turn off NF-kB activation, while neutrophils from young females were desensitized to apo F stimulation of PPARγ

**Fig. 7:**
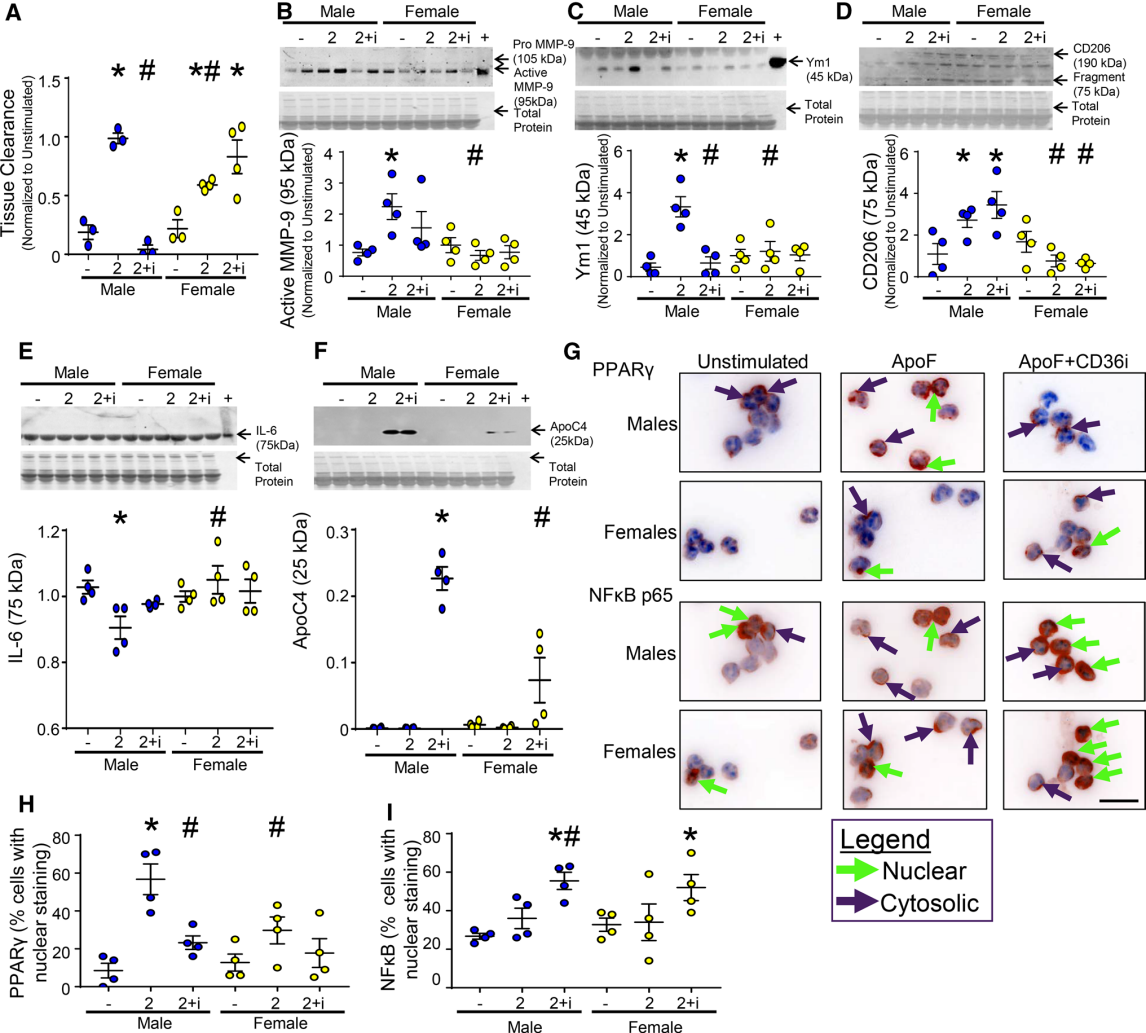
Apo F stimulation activated LXR/RXR signaling through PPARγ in males and not females. **a** Male neutrophils isolated from bone marrow and stimulated in vitro with Apo F (2 µg/mL) had increased tissue clearance capacity that was attenuated by CD36 inhibition (CD36i). **b** This was due to increased degranulation and release of active MMP-9. Neutrophils isolated from female mice had a blunted response to Apo F that was independent of CD36. Apo F stimulation increased (C) Ym1 and **d** CD206 fragment secretion in neutrophils from male mice, but not females. CD36i attenuated Ym1, but not CD206 release. **e** IL6 protein decreased in neutrophils isolated from males only, consistent with LXR/RXR activation. **f** CD36i induced secretion of ApoC-IV protein, an effect exaggerated in males compared to females. **g** Staining for nuclear factor (NF)-κB p65 or peroxisome proliferator-activated receptor (PPAR)γ (shown in red), with DAPI nuclear counterstain showed **h** Apo F stimulation increased PPARγ translocation in males but not females, a response regulated by CD36. **i** CD36i in the presence of Apo F activated NF-κB in both males and females. Scale bar = 25 µm, *n* = 4/group. Multiple group comparisons were analyzed by one-way ANOVA with Student–Newman–Keuls post-test. *p < 0.05 vs. unstimulated; ^#^*p* < 0.05 vs Apo F stimulated neutrophils from males

Neutrophil stimulation with apo F in vitro increased tissue clearance capacity (Fig. 7a) in both sexes, with males showing a greater response to the same apo F dose. This was opposite to what was observed for the ex vivo MI stimulated neutrophils, where female cells had higher clearance rates. This may be due to differences in culture conditions, as the ex vivo tissue clearance measurements were performed using cells, while the in vitro tissue clearance assay was performed using only the secretome of stimulated cells. Using only the secretome for in vitro studies allowed the effects of secreted proteins vs. cellular mechanisms to be dissected. Phorbol 12-myristate 13-acetate (PMA) stimulation had no effect on tissue clearance (*p *= 0.17 for males and *p *= 0.08 for females), demonstrating tissue clearance was not regulated through protein kinase C [63, 72].

CD36 regulates tissue clearance through phagocytosis and MMP-9 secretion [19]. In male but not female neutrophils, apo F stimulated MMP-9 release from tertiary granules (Fig. 7b), and CD36i attenuated this response. Tissue clearance in female neutrophils, therefore, relied more on NO and ROS production than degranulation of proteolytic enzymes, as with age female mice had increased NO and ROS production.

Apo F stimulated the release of two anti-inflammatory N2 markers, Ym1 and the 75 kDa proteolytic fragment of CD206 (Fig. 7c, d) [19]. The loss of Ym1 explained the tissue clearance capacity in male neutrophils, as Ym1 has been linked to neutrophil-mediated degradation of *N*-glycans [28]. CD36i attenuated the apo F-induced Ym1 increase while having no effect on CD206 fragment production.

Apo F stimulation decreased IL6 secretion through CD36 in male, but not female neutrophils (Fig. 7e). Interestingly, CD36i induced Apo C-IV secretion by male and female neutrophils (Fig. 7f), with a greater effect in males. Apo C-IV is downstream of LXR/RXR activation and is a regulator of lipid transport and metabolism [6, 55]. When CD36 was blocked, apo C-IV no longer trafficked into the cell and accumulated in the secretome. Reduced LXR/RXR activation explained why females secreted lower levels of apo C-IV and had a compensatory CD36 increase with age.

To determine the mechanism of apo F regulation of neutrophil activation, nuclear localization of NF-κBp65 and PPARγ was assessed (Fig. 7g). Apo F activated PPARγ in male neutrophils (Fig. 7h), consistent with inhibition of IL6 and induction of Ym1 and CD206 [46, 64, 65]. This effect was attenuated by CD36i. Apo F stimulation did not activate PPARγ in female neutrophils; as a result, MMP-9, Ym1, CD206, and IL6 were not induced. TSP-1 signaling was upregulated in the infarct issue of post-MI females (Fig. 2), which may have resulted in upregulation of CD36, AMPK, and p38 MAPK signaling as compensatory mechanisms [12, 38, 69]. AMPK inhibits PPARγ activation [73], which links TSP-1 signaling to attenuation of LXR/RXR signaling.

While Apo F stimulation alone did not affect NF-κBp65 nuclear localization, CD36i induced NF-κBp65 translocation to the nucleus in both male and female neutrophils (Fig. 7i). As PPARγ is a negative regulator of NF-κB activation [1, 74], the decrease in PPARγ with CD36i would activate NF-κB. This is consistent with the tissue array results, which showed both males and females deactivated PPAR signaling early post-MI to activate NF-κB. Increased NF-κBp65 leads to an elevated pro-inflammatory response; thus activation of LXR/RXR signaling through apo F and PPARγ stimulated resolution of neutrophil-mediated inflammation in both sexes.

### Loss of LXR/RXR signaling in women with MI correlated with increased risk of heart failure

To assess translational relevance of our findings in mice, we analyzed plasma samples from the Jackson Heart Study. Of the 60 individuals that met inclusion criteria, 15 experienced subsequent heart failure hospitalization after visit 2 (*n *= 3 men/12 women). This 25% post-MI heart failure development rate is consistent with incidence rates in past clinical reports [24, 29, 68, 79]. Women had a higher incidence of heart failure after visit 2 (447 events per 100 person-years) compared to men (56 events per 100 person-years). In addition, women who developed heart failure were older (73 ± 9 vs 58 ± 9 years) and had higher systolic blood pressure (146 ± 24 vs 108 ± 8 mmHg) and lower hemoglobin A1C levels (7.3 ± 2.0% vs 9.2 ± 0.9%) compared to men who progressed to heart failure (Online Table 3).

**Fig. 8:**
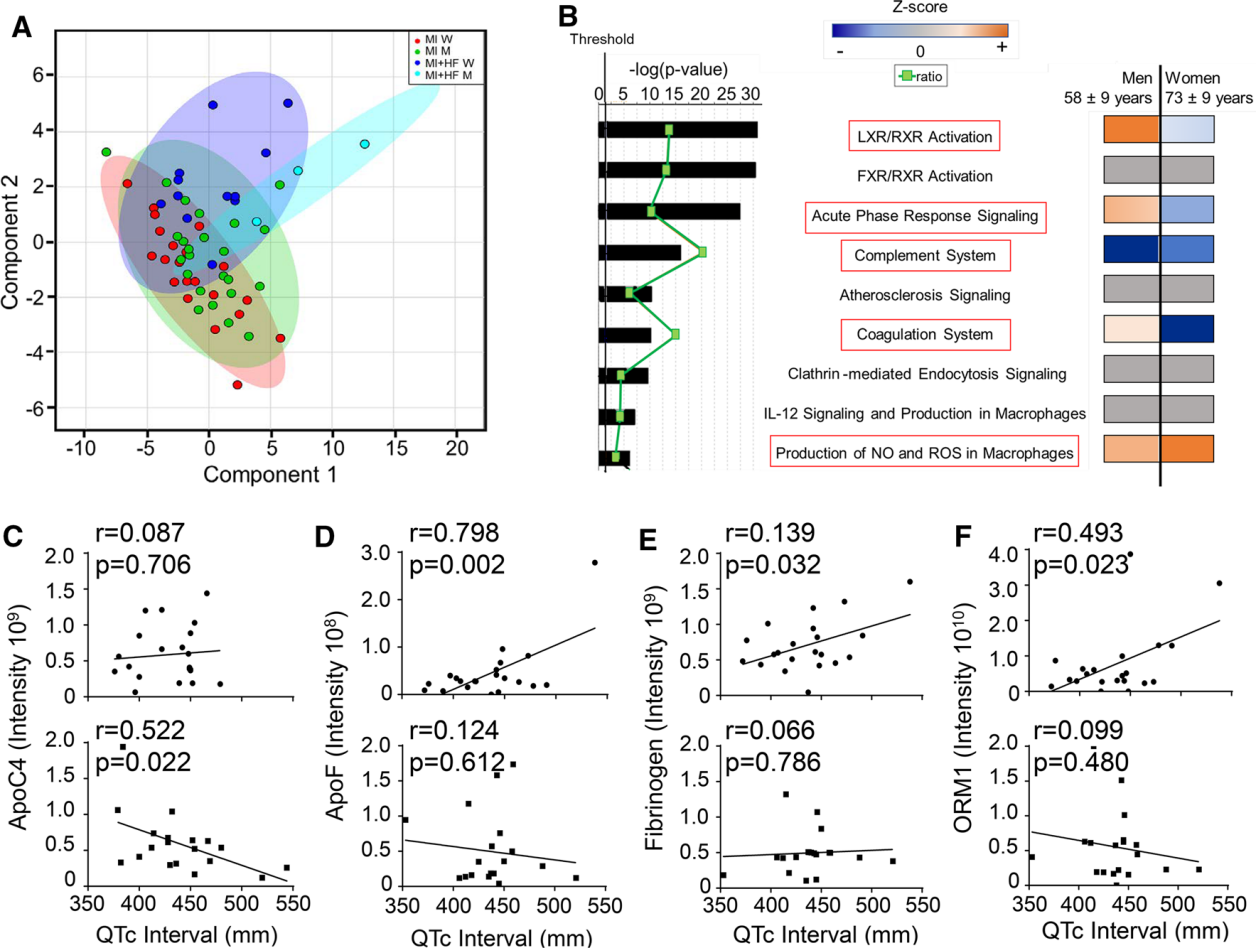
Gender differences in humans revealed by partial least squares discriminant analysis and canonical pathway analysis of glycoproteomic profiles. **a** Glycoprotein signatures of men and women in the MI (no heart failure) groups overlapped, while men and women in MI + heart failure groups showed distinct cluster distributions (JHS cohort). **b** Men and women differed in pathways upregulated or downregulated in response to heart failure. Both genders had upregulation of production of nitric oxide (NO) and reactive oxygen species (ROS) and downregulation of the complement system. Men with heart failure had upregulation of the LXR/RXR and acute phase response pathways, whereas women with heart failure had down regulation of these pathways and the coagulation system. **c** ApoC-IV (an LXR/RXR pathway member) negatively correlated with QTc interval in women, but not men. **d** Apo F, **e** fibrinogen, and **f** orosomucoid 1 (ORM) positively correlated with QTc interval in men only. MI + heart failure data were normalized to MI only to calculate fold change for each sex. The most statistically significant canonical pathways identified are listed according to p value (bars), with the ratio of the number of pathway proteins identified in glycoproteomic data over the total number of proteins in that pathway (green squares). Multiple-testing corrected p values were calculated using the Benjamini–Hochberg method. The threshold line corresponds to a *p* value of 0.05

Glycoproteomic analysis identified 200 peptides (Online Table 4) belonging to 88 unique proteins. The mass spectrometry proteomics data have been deposited to the ProteomeXchange Consortium via the PRIDE partner repository with the dataset identifier PXD009870 (http://www.ebi.ac.uk/pride/archive/) [80]. By partial least squares discriminant analysis, participants with MI who later presented with adjudicated heart failure hospitalization showed a distinct separation of the sexes based on the lack of ellipse overlap (Fig. 8a). For participants with stable MI, there was a lack of sex effect based on the overlap of ellipses.

Glycoproteomic data from the heart failure group was normalized to respective sex MI only groups to identify early upregulation of glycoproteins that predict later heart failure development. By Ingenuity Pathway Analysis, pathways associated with MI response and not linked to heart failure development included the farnesoid X receptor (FXR)/RXR, atherosclerosis, clathrin-mediated endocytosis, and IL12 signaling and production pathways, all of which had a Z score of zero. Glycoproteins associated with heart failure mapped to the following pathways: LXR/RXR, acute phase response, complement system, coagulation system, production of NO and ROS pathways. Similar to the aged mice, pathways upregulated in men and downregulated in women included LXR/RXR (*p *= 1.36 × 10^−29^), acute phase response (*p *= 1.14 × 10^−26^), and coagulation (*p *= 9.09 × 10^−10^; Fig. 8b). The LXR/RXR and acute phase response pathways accounted for 22 of the 88 proteins (25%). Pathways in common to both men and women included complement system (downregulated) and production of NO and ROS (upregulated).

To assess if loss of LXR/RXR or acute phase signaling could facilitate heart failure development, we correlated the proteins identified in these pathways with QT interval measurements taken at visit 3. Longer QTc intervals associate with poor post-MI prognosis, including increased mortality and heart failure [2, 47]. Apo C-IV negatively correlated with longer QTc intervals in women, but not in men (Fig. 8c), while Apo F, fibrinogen, and orosomucoid 1 positively correlated with longer QTc intervals in men, but not women (Fig. 8d–f). Consistent with our in vitro neutrophil results, loss of LXR/RXR signaling in women decreased apo C-IV, increased QTc intervals, and facilitated the development of heart failure by failing to inhibit NF-κB and thus prolonging the inflammatory response. This is consistent with previous work by Mayr et al. who demonstrated a strong association between very low-density lipoprotein-associated apolipoproteins including C-II, C-III, and E with incident cardiovascular disease [62].

### Interleukin 6 acted as a downstream regulator

**Fig. 9:**
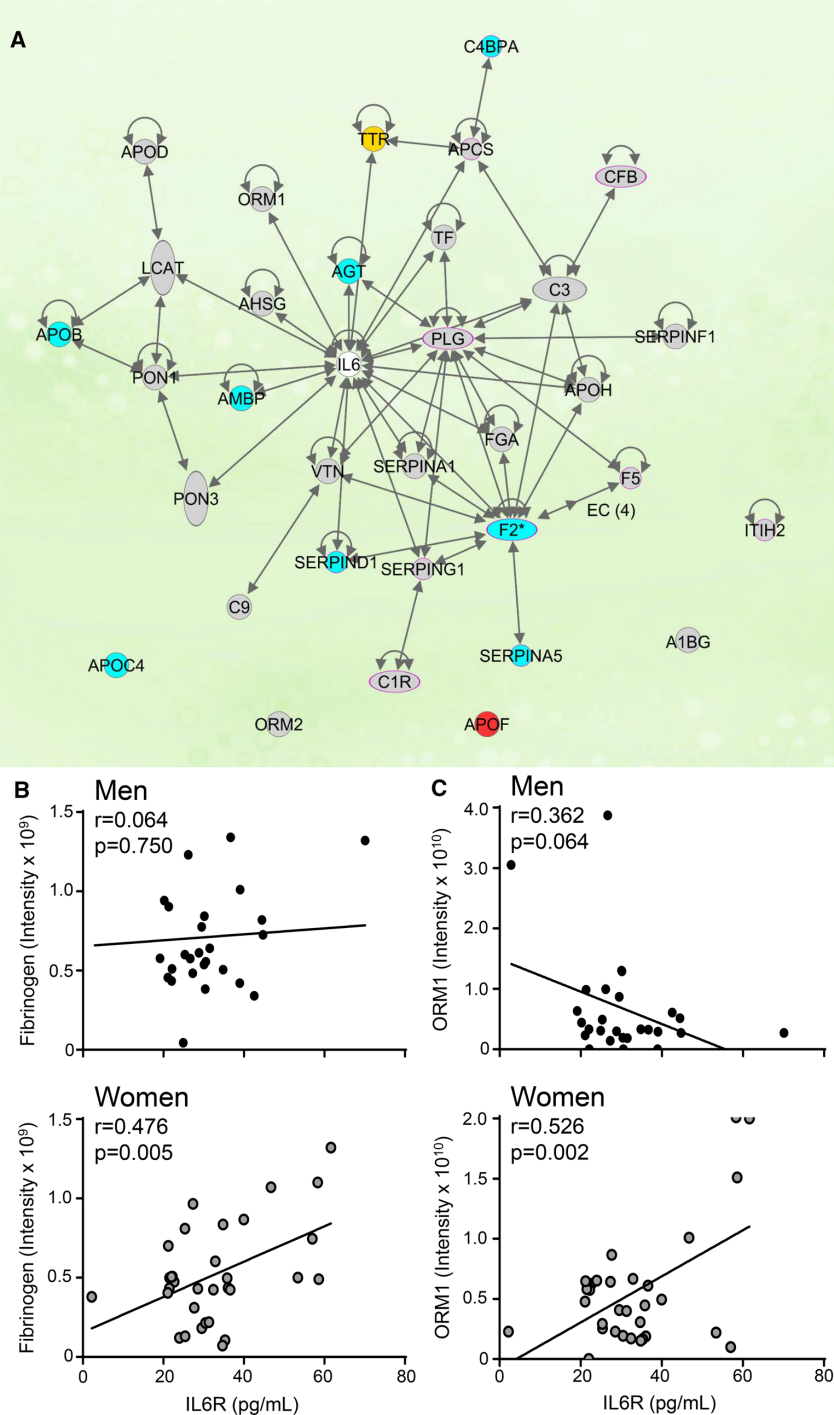
IL6 is a central component in the activation of the LXR/RXR, acute phase response, and coagulation pathways. **a** Of the 88 proteins identified by glycoproteomics, 22 (25%) were in the IL6 network. Red—proteins upregulated in both men and women with later heart failure compared to MI patients without later heart failure. Yellow— proteins upregulated in women with later heart failure but not in men with later heart failure. Blue—proteins upregulated in men with later heart failure, but not women with later heart failure. Grey—proteins neither upregulated nor downregulated in men or women with later heart failure. IL6 receptor correlated with **b** fibrinogen and **c** ORM1 in women but not men, *n* = 27 men (24 MI and 3 MI + heart failure) and 33 women (21 MI and 12 MI + heart failure)

Similar to mice, IL6 was a downstream regulator of sex differences during development of heart failure, as 28 of the 88 glycoproteins identified (32%) interact with IL6 (Fig. 9a). Of the 28 proteins within the network, apo C-IV, angiotensinogen, alpha-1-microglobulin, complement 4b-binding protein alpha, thrombin, serpinD1, serpinA5, and apo B were elevated in men who developed heart failure, but not in women. Women who went on to develop heart failure had significant upregulation of transthyretin. IL6 stimulates angiotensinogen, serum amyloid P, fibrinogen, and orosomucoid-1 and inhibits transthyretin [34, 41, 42] signifying men who developed heart failure had increased IL6 activity, whereas women with heart failure had decreased IL6 activity. Apo F, which acts upstream of the LXR/RXR pathway, [21] was increased in both men and women with heart failure. Fibrinogen and orosomucoid 1 were elevated and correlated with plasma IL6 receptor in women, but not men (Fig. 9b, c). These results revealed early plasma indicators of later heart failure development, with profiles distinguishing men and women.

## Discussion

**Fig. 10:**
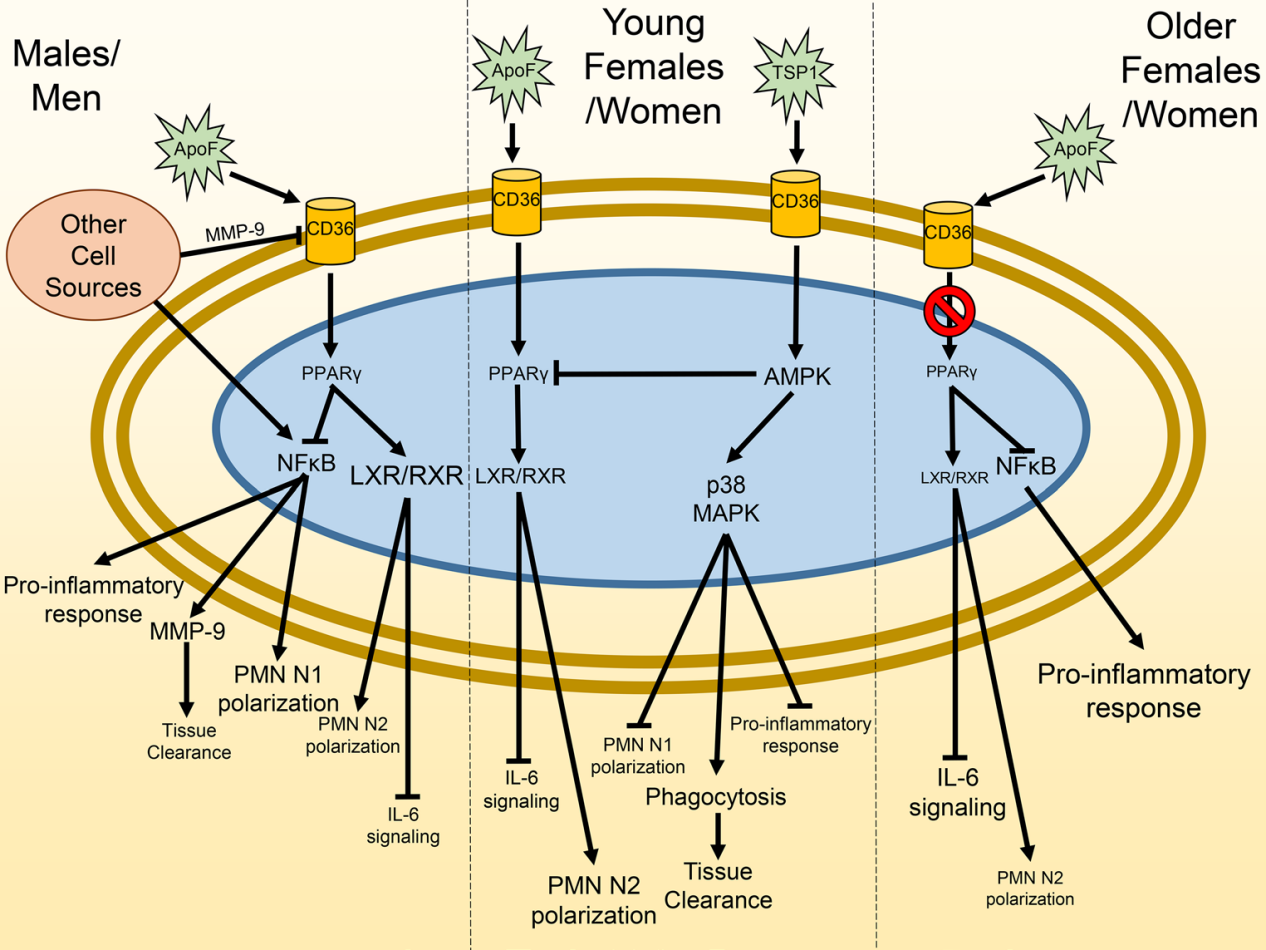
Summary of sex-dependent differences in cardiac remodeling after myocardial infarction (MI). After MI, men/males stimulate PPARγ and LXR/RXR signaling as an attempt to promote resolution of inflammation. Women/females use both LXR/RXR and thrombospondin signaling to promote decreased inflammation and increased necrotic tissue clearance. With age, females are desensitized to LXR/RXR and PPARγ activation, resulting in increased IL6 signaling and a loss of protection. The network was mapped based on experimental data as well as literature

The goal of this study was to investigate post-MI sex differences in LV remodeling progression to heart failure. The most salient findings were: (1) in vivo, young female mice were more efficient than young males or old mice of either sex at resolving the inflammatory response through differences in neutrophil physiology; (2) in vitro, neutrophils from males stimulated with apo F activated the LXR/RXR pathway through CD36 and PPARγ activation to turn off NF-kB activation, while neutrophils from young females were desensitized to apo F stimulation of PPARγ and relied on TSP-1 stimulation of CD36 to upregulate AMPK and stimulate phagocytic potential; (3) with age, female mice were desensitized to LXR/RXR signaling, resulting in enhanced IL-6 activation; and (4) in humans, men and women remodeled using distinct pathways (men upregulated LXR/RXR whereas women downregulated LXR/RXR) to arrive at a similar pathophysiological end point (i.e., heart failure). Our data revealed the sex-dependent mechanisms whereby mice and humans with MI progress to heart failure (Fig. 10). To our knowledge, this is the first report to dissect the distinct neutrophil signaling pathways stimulated after MI to activate progression to heart failure.

The major strengths of this study include the use of both mice and human data to provide a cross-translational perspective, the evaluation of human MI samples before heart failure was diagnosed, and the use of a convergence approach using both genomics and proteomics in plasma, LV tissue, and isolated neutrophils to provide a comprehensive mechanistic evaluation. Our results highlight the strong agreement at gene and protein levels for the pathways most involved in the post-MI progression to heart failure (Online Table 5).

Although sex differences in cardiovascular disease are caused in part by environmental or social differences such as occupational hazards, habits, and social stresses, we focused here on biological factors that regulate cardiac wound healing. This study used a population-based cohort of middle aged and older adults of African Ancestry in a single community, which strengthened the translational impact of our findings. As sample sizes could not be controlled given the retrospective study design, the n for the male heart failure group was small and glycoproteomics was measured at a single time point, which varied in its temporal relation between MI and onset of heart failure. Of note, the incidence of heart failure was 25%, which is similar to what larger clinical studies have shown [24, 29, 68, 79]. Comparison of our findings with a larger prospective cohort and including additional ethnicities/races, regions/countries, and ages is warranted.

Sex differences in inflammation have been shown in animal models and humans. Men have elevated leukocyte-mediated inflammation in atherosclerotic plaques, consistent with the pro-inflammation status seen in the post-MI LV [20]. In contrast, young women have a more temperate inflammatory response. Our data revealed that neutrophils isolated from males remove necrotic tissue through CD36-dependent degranulation of MMP-9. Females, on the other hand, removed necrotic tissue by secreting proteins, in addition to cellular mechanisms including phagocytosis. Neutrophils in young females were more efficient at removing necrotic tissue than neutrophils isolated from young males. Reduced neutrophil numbers observed in female hearts after MI also protects from collateral damage incurred by neutrophil-derived mediators. While both neutrophils and macrophages use LXR/RXR signaling and are equally responsible for adverse post-MI remodeling [58, 67, 78], the neutrophil difference was prominent. Future studies that investigate the role of other immune cells, including macrophages and T and B lymphocytes, are needed to fully understand all sex differences in cardiovascular disease pathology. In a mouse model of sepsis, females exhibited dampened inflammation and less tissue injury due to heightened leukocyte sensitivity to infectious and injurious stimuli compared to male mice [71]. In our study, the sex difference in inflammation diminished with age, as female mice (11–36 months-old) and women both showed reduced LXR/RXR pathway signaling.

LXR/RXR signaling was a top ranked canonical pathway associated with post-MI development of heart failure in our human sample set, highlighting the significance of this pathway. LXR/RXR signaling regulates inflammation, cholesterol homeostasis, and lipid and glucose metabolism [8]. After ischemic injury, the LXR/RXR pathway decreases cardiomyocyte hypertrophy and promotes cell survival, modulates myocardial metabolism, promotes angiogenesis, and attenuates fibrosis [8]. Estrogen attenuates and antagonizes the expression of LXR and its transcriptional activity [11, 25, 44]. Interestingly, our data showed that aged females had a loss of LXR/RXR activation, demonstrating in neutrophils the effects that we observed are most likely independent of hormonal influences and more likely due to X chromosome actions.

Apo F indirectly regulates CD36 by binding to high-density lipoproteins and is a known activator of the LXR/RXR pathway [43]. Our study indicated Apo F regulated neutrophil mediated-tissue clearance in males by increasing secretion of MMP-9. Interestingly, with CD36i, MMP-9 secretion was attenuated in males, indicating neutrophil secretion of MMP-9 is independent of NF-κB activation. Female neutrophils, however, regulated tissue clearance independent of CD36 and MMP-9. Our data indicate females remove necrotic tissue through secretion of both NO and ROS as well as through phagocytosis. In turn, this would result in better removal of necrotic tissue without excessive damage to the myocardium.

Our data reveal different roles played by PPARγ in women versus men. A similar paradigm occurs in T cells, where PPARγ inhibits activation in female, but not male cells [61]. Neutrophils from females did not rely on PPARγ and instead activated AMPK through TSP activation of CD36. Our understanding of the cross-talk from PPARγ to AMPK remains controversial, as AMPK activation is either a PPAR-γ-dependent or independent process depending on the study [57]. AMPK also inhibits PPARγ activation in other disease models [73] representative of bi-directional cross-talk, which would explain why in vitro Apo F stimulation did not activate PPARγ in females but still triggered a physiological response (e.g., tissue clearance).

After MI, NF-κB was activated in males to stimulate pro-inflammatory N1 neutrophils to release MMP-9. During heart failure development, Apo F increased in an attempt to protect the myocardium by activating LXR/RXR signaling through CD36 and PPARγ activation. When CD36 was inhibited or cleaved by MMP-9, [19] PPARγ could no longer inhibit NF-κB activation, leading to increased N1 polarization and decreased N2 polarization. In vivo, young females activated the NF-κB pathway to a lesser extent and relied on TSP-1 activation of the AMPK pathway. With age, females lost their ability to activate the LXR/RXR pathway, resulting in increased NF-κB activation despite elevated Apo F levels. A loss of LXR/RXR signaling in older female mice may, therefore, be detrimental by preventing a post-MI inflammation resolution response, resulting in prolonged exposure to pro-inflammatory cytokines such as IL6. Our study provides the first evidence that the myocardium of older men and women remodel after MI using divergent pathway activation to arrive at a common pathophysiological end point.

The comparison between human and mice responses to MI shows that while there are distinct species attributes, there exists significant commonality in the post-MI response (Online Table 6). When gene expression was compared across human and mouse models of burn, sepsis, and trauma, researchers found significant species overlap among conditions and pathways [75]. This was especially true for genes involved in the innate immune response, cytokine signaling in immune system, and lymphocyte differentiation. Our findings, therefore, indicate that cross-species comparisons can help to identify salient pathways. The alignment between gene and protein and between plasma and LV infarct tissue was also well preserved, signifying that combining approaches can provide a powerful tool to increase the signal to noise ratio and hone in on the most informative signaling pathways.

One strength to our study is that we used the non-reperfused MI mouse model in both young and old animals, which provides robust remodeling endpoints (i.e., ventricular rupture and mortality) [39, 59]. The majority of MI patients who present to the emergency department undergo reperfusion and are optimally treated with aspirin, angiotensin converting enzyme inhibitors, beta blockers, and statins and are not reflected by the permanent occlusion model. Our model represents the 40% of MI patients who either do not receive timely reperfusion due to late presentation or confounding issues or go on to develop heart failure. Although rupture rates for human patient populations are difficult to standardize and compare across studies, clinical evidence supports the concept that older female patients are more likely to rupture than males [30]. We evaluated LV tissue and plasma in mice, while in the human cohort only plasma samples were evaluated. Thus, all healing patterns observed in the mouse model were interpreted with caution when comparing to the population dataset.

In conclusion, inflammation resolution was a critical component of MI remodeling that occurred under sex-dependent mechanisms. Our results highlight the need to not only understand mechanisms that dampen but also mechanisms that turn off inflammation [27, 33, 35, 36].

## Electronic supplementary material

Below is the link to the electronic supplementary material.

Supplementary material 1 (tiff 2852 kb)

Supplementary material 2 (tiff 7238 kb)

Supplementary material 3 (docx 2585 kb)

Supplementary material 4 (xlsx 221 kb)
