# Supplementary figures and images for: LXR/RXR signaling and neutrophil phenotype following myocardial infarction classify sex differences in remodeling
Source: Basic Res Cardiol. 2018 Aug 21;113(5):40. doi: 10.1007/s00395-018-0699-5 (PMC6105266; doi:10.1007/s00395-018-0699-5)

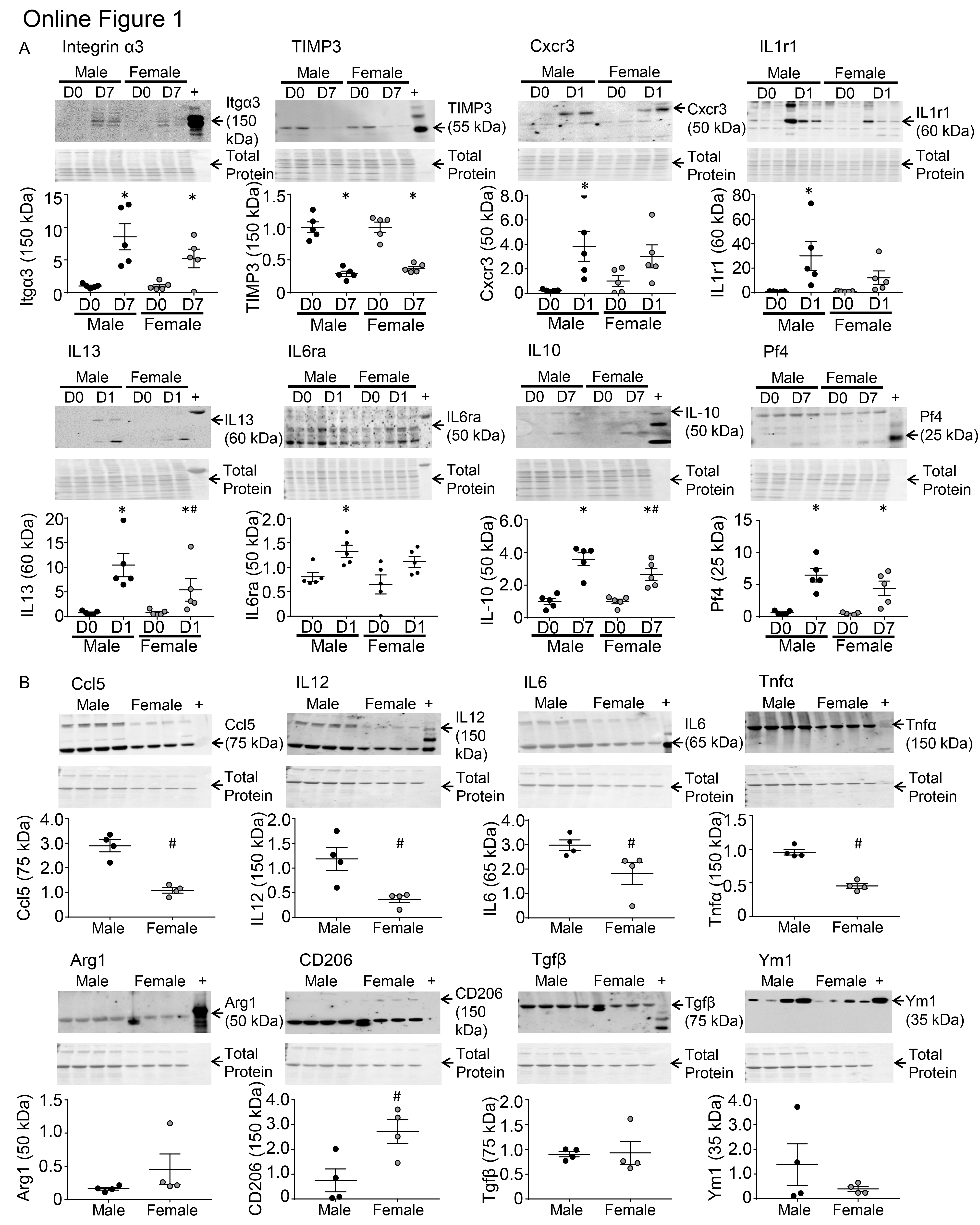

Supplement: Supplementary file 1 — Supplementary material 1 (tiff 2852 kb) [file 395_2018_699_MOESM1_ESM.tif]

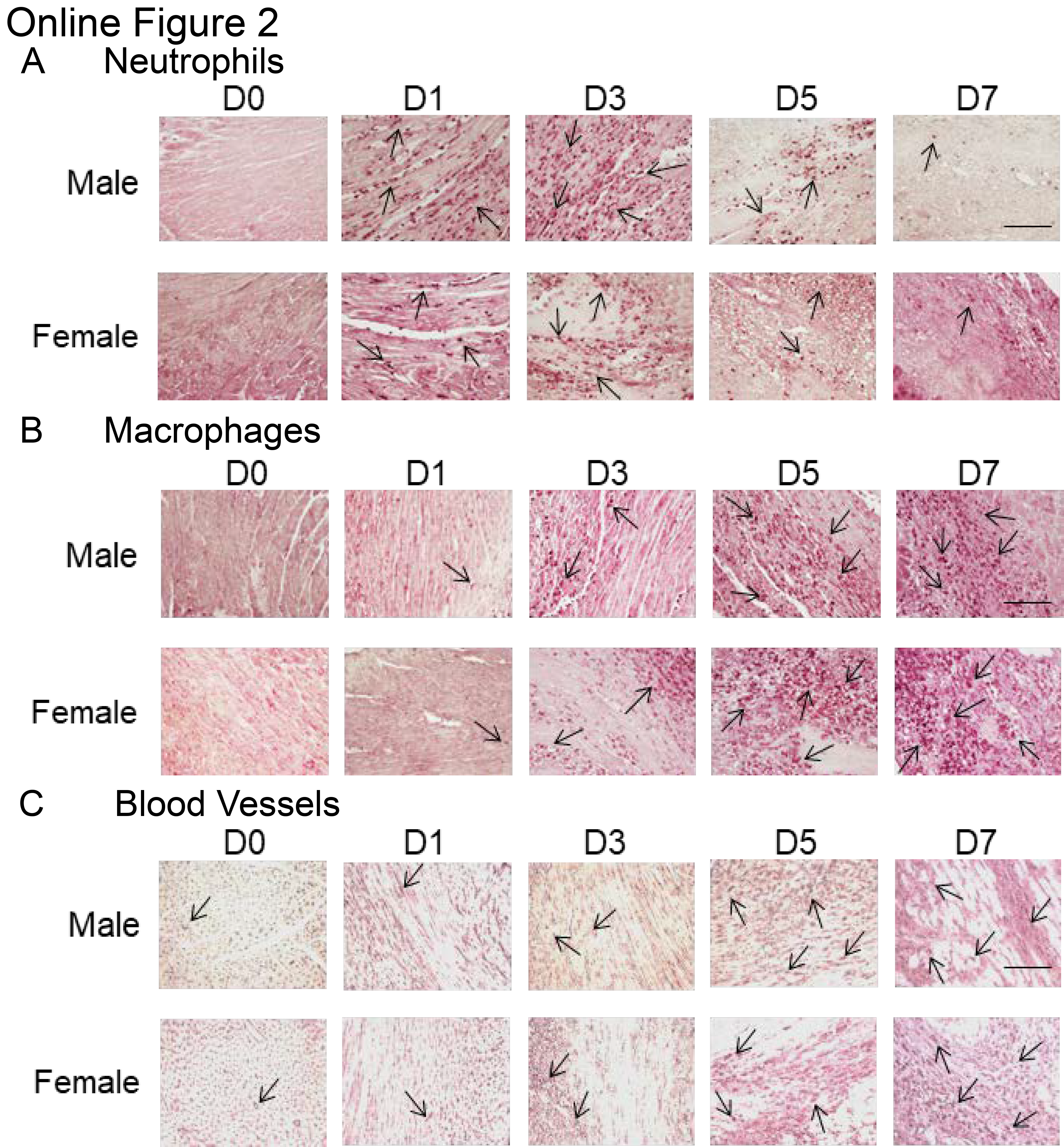

Supplement: Supplementary file 2 — Supplementary material 2 (tiff 7238 kb) [file 395_2018_699_MOESM2_ESM.tif]
