# Supplementary material for: LXR/RXR signaling and neutrophil phenotype following myocardial infarction classify sex differences in remodeling
Source: Basic Res Cardiol. 2018 Aug 21;113(5):40. doi: 10.1007/s00395-018-0699-5 (PMC6105266; doi:10.1007/s00395-018-0699-5)
Supplement: Supplementary file 3 — Supplementary material 3 (docx 2585 kb) [file 395_2018_699_MOESM3_ESM.docx]

**Title: LXR/RXR Signaling and Neutrophil Phenotype Following Myocardial Infarction**

**Classify Sex Differences in Remodeling**

Kristine Y DeLeon-Pennell^1,2*^, Alan J Mouton^1^, Osasere K Ero^1^, Yonggang Ma^1^,

Rugmani Padmanabhan Iyer^1^, Elizabeth R Flynn^1^, Ingrid Espinoza^3^, Solomon K. Musani^4^, Ramachandran S Vasan^5^, Michael E Hall^1,4,6^, Ervin R Fox^4,6^, and Merry L Lindsey^1,2*^

^1^Mississippi Center for Heart Research, Department of Physiology and Biophysics, UMMC, Jackson, MS; ^2^Research Service, G.V. (Sonny) Montgomery Veterans Affairs Medical Center, Jackson, MS; ^3^Department of Preventive Medicine and Cancer Institute, UMMC, Jackson, MS; ^4^Jackson Heart Study, UMMC, Jackson, MS; ^5^Preventive Medicine and Epidemiology and Cardiology, Department of Medicine, Boston University School of Medicine; ^6^Division of Cardiology, UMMC, Jackson, MS

***Address for Correspondence:**

| Kristine Y. DeLeon-Pennell, Ph.D. or | Merry L. Lindsey, Ph.D. |
| --- | --- |
| (phone) 843-789-6839  (fax) 601-984-1817  Email: [deleonky@musc.edu](mailto:deleonky@musc.edu) | (phone) 601-815-1329  (fax) 601-984-1817  Email: [mllindsey@umc.edu](mailto:mllindsey@umc.edu) |
| Department of Physiology and Biophysics  University of Mississippi Medical Center  2500 North State St., Jackson, MS 39216-4505, USA | |

**Supplemental Materials:**

**Material and Methods**

**mHART Database.** Extensive details on the mHART database have been published previously.[5] We collected temporal results ranging from day 0 unoperated controls to day 28 after myocardial infarction (MI). Each mouse received a unique identification number when entered into the colony, which allowed examination of variables across platforms. All mice were obtained from The Jackson Laboratory or from in-house breeding colonies. All animal use was approved by the local institutional animal care and use committee. Data was collected by multiple investigators from one lab in a systematic and uniform manner, with investigators blinded to groups during acquisition and analysis.

All data underwent at least two rounds of quality assessments to certify data was precise and accurate- one at the initial time of collection and analysis and one at the time of inclusion into the database. Data first underwent internal checks for consistency of measurements within groups, and any potential outliers were re-analyzed by two or more investigators in a blinded manner. If inconsistent results were observed, data underwent an additional round of cleaning steps to ensure quality control and data validation. By ensuring data infrastructure was stable and easily accessible, we were able to derive new data-driven hypotheses. The database included results on echocardiography, MI surgery and necropsy, immunohistochemistry, real time RT^2^-PCR, and plasma proteomic profiling. Methods for acquiring and analyzing these results are provided below.

**Echocardiography.** Transthoracic echocardiography was acquired using either a Vevo770 or 2100^TM^ system (VisualSonics, Toronto, Ontario, Canada) with a 30 MHz image transducer as previously described.[7] Mice were anesthetized with 0.5–2.0% isoflurane in 100% oxygen. Electrocardiogram, heart rate, and body temperature were monitored during the imaging procedure. Measurements were taken from the left ventricle (LV) parasternal long axis (B-mode) and short axis (M-mode) views. For each echocardiographic variable, three images from consecutive cardiac cycles were measured and averaged. All images were acquired at heart rates >400 bpm for physiologically relevant measurements. Echocardiography data was normalized to LV mass to remove an effect of size differences between sexes.

**Permanent coronary artery ligation surgery**. All mice were kept in the same room in a light-controlled environment with a 12:12 hour light-dark cycle and with free access to standard mouse chow and water. Mice were anesthetized with 1.5–2.0% isoflurane in 100% oxygen during the MI surgery, which was performed as described previously.[14] Mice were given buprenorphine injection (0.05-0.1 mg/kg, subcutaneous or intraperitoneal) before MI to alleviate post-operative stress and pain.

**Tissue harvest and infarct area evaluation.** Necropsy was performed at the termination time point as described previously. For sacrifice, mice were anesthetized with 0.5-2.0% isoflurane in an oxygen mix. Heparin was administered (i.p., 4U/g body weight) and plasma was collected 5 min later. The hearts were flushed with cardioplegic solution (NaCl, 69 mM; NaHCO3, 12 mM; glucose, 11 mM; 2,3-butanedione monoxime, 30 mM; EGTA, 10 mM; Nifedipine, 0.001 mM; KCl, 50 mM) to arrest the heart in diastole. The hearts were excised and the LV and right ventricle were separated and weighed individually. The LV was sliced into apex, middle, and base sections, stained with 1% 2, 3, 5- triphenyltetrazolium chloride (TTC, Sigma), and photographed for evaluation of infarct area using Photoshop (Adobe). Values are presented as percentage of infarct area to total LV area. For the apex and base sections, the LV infarct region (LVI) was separated from non-infarcted remote region (LVC), individually snap frozen, and stored at -80°C. The lung weights and tibia lengths were collected.

**Immunohistochemistry.** The LV middle section was fixed in 10% zinc formalin (Fisher Scientific), paraffin-embedded, and sectioned for histological examination as described previously.[7] Heat mediated antigen retrieval (Target retrieval solution, Dako) was performed to expose antigen epitopes. Sections were blocked with rabbit blocking serum of Vectastain elite ABC Kit (Vector Laboratories, Marion, IA, USA). Tissue was incubated in a primary antibody specific for macrophages (Mac-3, Cedarlane CL8943AP; 1:100) or neutrophils (PMNs; anti-neutrophil mouse monoclonal, CedarlaneCL8993AP; 1:100) at 4°C overnight. After incubation, the sections were incubated with the respective secondary antibodies. Positive staining was visualized by HistoMark Black (KPL 54-75-00) and eosin was used as a counterstain. Picrosirius red (PSR) staining was used to examine collagen density. Sections were stained with biotinylated GSL-I (100 μg/ml) followed by avidin-biotinylated enzyme complex and 3,3′-diaminobenzidine chromogen to visualize endothelial cells. Images were captured at 40X magnification with Image-Pro software (Media Cybernetics, Bethesda, MD, USA) and quantification was calculated as percentage of positive stained area to total area.

**Real Time RT^2^-PCR.** RNA extraction was performed using TRIzol® Reagent (Invitrogen Life Technologies, Grand Island, NY, USA) according to manufacturer instructions as described previously.[7] RNA levels were quantified using the NanoDrop ND-1000 Spectrophotometer (Thermo Scientific, Waltham, MA, USA). Reverse transcription of RNA (0.4 µg) was performed using the RT^2^ First Strand Kit (Qiagen, Valencia, CA, USA). Real-time RT^2^-PCR gene array for inflammatory cytokines and receptors (Qiagen, Valencia, CA, USA) and for extracellular matrix (ECM) and adhesion molecules (Qiagen, Valencia, CA, USA) were performed to quantify gene expression levels. The gene levels were normalized to the reference gene hypoxanthine guanine phosphoribosyl transferase 1 (*Hprt1*). The experiments were performed according to the MIQE guidelines with one exception. Hprt1, was the only reference gene showing no change in expression after MI (GusB, Hsp90ab1, Actb, and Gapdh significantly changed post-MI).[8]

**Plasma proteomic profiling.** For plasma proteomic profiling, blood was collected from the common carotid artery as described above and immediately centrifuged for collection of plasma. Complete proteinase inhibitor cocktail (1x; Roche, Indianapolis, IN) was added to the plasma. Samples (100 uL) were sent to Rules Based Medicine (Austin, TX) for multi-analyte proteomic profiling.[3, 4]

**PMN phenotyping.** Total RNA was isolated from PMNs using TRIzol reagent (15596026, Life Technologies) plus total RNA purification kit (12183018A, Life Technologies). cDNA was synthesized using a High-Capacity RNA-to-cDNA Kit (4837406, Life Technologies). The expression levels of Ccl3 (Mm00441259_g1), Ccl5 (Mm01302427_m1), Il1b (Mm01336189_m1), IL6 (Mm00843434_s1), IL12a (Mm00434165_m1), Tnfa (Mm00443258_m1), Arg1 (Mm00475988_m1), CD206 (Mm00485148_m1), IL10 (Mm00439614_m1), Tgfb1 (Mm01178820_m1), Ym1 (Mm00657889_mH), Mmp8 (Mm00439509_m1), and Mmp9 (Mm00442991_m1), were measured with the Taqman gene expression master mix (4369016, Life Technologies). Hprt1 (Mm01545399_m1) was used as the reference gene.

PMNs isolated from the LV at day 1 post-MI were also evaluated for protein expression. Protein was extracted from the cells by dissolving in reagent type 4 (Sigma; 7 M urea, 2 M thiourea, 40 mM Trizma^®^ base and the detergent 1% C7BzO, 50 μL per 1x10^5^ cells). Total protein (2 µg for cells) was separated on 4-12% Criterion™ XT Bis-Tris gels (Bio-Rad), transferred to a nitrocellulose membrane (Bio-Rad), and stained with MemCode™ Reversible Protein Stain Kit (Thermo Scientific) to verify protein concentration and loading accuracy. Membranes were blocked with 5% nonfat milk (Bio-Rad) and incubated in primary antibodies overnight [IL6 (Cell Signaling 12912s; 1:1000), CCL5 (Abcam ab189841; 1:1000), IL12a (Abcam ab131039; 1:1000), TNF alpha (Novus NB600-587; 1:1000), Arg1 (R&D af5868; 1:1000), CD206 (Abcam ab64693; 1:1000), TGF beta (Abcam ab92486; 1:1000), Ym1 (Abcam ab93034; 1:1000)]. Relative expression was calculated as the densitometry of the protein of interest divided by the densitometry of the entire lane of the total protein stained membrane. Protein levels were quantified by densitometry using the IQ-TL image analysis software (GE Healthcare, Waukesha, WI).

**Protein extraction and analysis.**  Protein was extracted from LV infarct tissue by homogenizing the samples sequentially in phosphate buffered saline (PBS) with 1x protease inhibitor cocktail (16 μL per mg tissue, soluble protein fraction), followed by protein extraction reagent type 4 (Sigma; 7 M urea, 2 M thiourea, 40 mM Trizma^®^ base and the detergent 1% C7BzO, 15 μL per mg tissue) with 1x protease inhibitor cocktail (insoluble protein fraction).

Protein concentrations were determined by the Quick Start™ Bradford Protein Assay (Bio-Rad). Total protein (10 µg for tissue and 5 uL of media for secretome) was separated on 4-12% Criterion™ XT Bis-Tris gels (Bio-Rad), transferred to a nitrocellulose membrane (Bio-Rad), and stained with MemCode™ Reversible Protein Stain Kit (Thermo Scientific) to verify protein concentration and loading accuracy.

For immunoblots using tissue, membranes were blocked with 5% nonfat milk (Bio-Rad), followed by overnight incubation with an antibodies against primary antibody [IL6ra (Abcam ab83053; 1:1000), Cxcr3 (R&D MAB1685; 1:1000), IL13 (R&D MAB413; 1:2500), IL1r1 (Abcam ab106278; 1:500), tissue inhibitor of matrix metalloproteinase-3 (TIMP-3, Abcam ab39184; 1:1000), IL10 (Abcam ab33471; 1:500), integrin α3 (R&D AF2787; 1:1000), CD36 (Novus Biologicals NB400-144; 1:1000), and apolipoprotien F (Abcam ab77452; 1:1000)], Toll-like receptor 4 (TLR4) (Biolegend 117608, 1:1000, secondary antibody (anti-mouse Cell Signaling, 7076s, 1:3000, anti-sheep Abcam, ab6747 1:3000, anti-rabbit Vector, PI-1000, or anti- rat Vector, PI-9400, 1:5000), and detected with ECL Prime Western Blotting Detection Substrate (Amersham). For validation experiments, integrin α3, tissue inhibitor of metalloproteinase (TIMP)-3, Cxcr3, IL1r1, IL13, and IL6ra were chosen as they showed biggest difference between male and female mice post-MI. interleukin (IL)10 and Pf4 were chosen as negative controls because they were not significantly different at the gene level.

For evaluation of proteins secreted by bone marrow PMNs after ApoF stimulation, membranes were blocked in 15% FBS, 5% nonfat milk in TPBS and incubated in primary antibodies [IL6 (Cell Signaling 12912s; 1:1000), apolipoprotein C-IV (Abcamab54804; 1:1000), MMP-9 (Abcamab38898; 1:1000), CD206 (Abcam ab64693; 1:1000), Ym1 (Abcam ab93034; 1:1000)]. Relative expression was calculated as the densitometry of the protein of interest divided by densitometry of the entire lane of the total protein stained membrane. Protein levels were quantified by densitometry using the IQ-TL image analysis software (GE Healthcare, Waukesha, WI).

***In vitro* PMN stimulation.** PMNs were isolated from the bone marrow of no MI naive mice as described previously.[9] Cells (5x10^5^/condition) were stimulated with 0, 0.5, 1.0, or 2.0 µg/mL of apolipoprotein F (ApoF; Abcam ab116968) for 15 min at 37˚C. The peak response was seen at 2 µg/mL ApoF, and this concentration was used for the remaining experiments. PMNs were incubated with or without CD36 blocking antibody (CD36i; Millipore MABF956) to assess if signaling. PMNs were also stimulated with phorbol myristate acetate (PMA; 100 µM).

**Tissue Clearance Capacity.** To assess tissue clearance capacity of PMNs, cells were isolated from day 1 infarcts by tissue enzymatic digestion to single cells and plated in duplicate (1.0x10^5^ cells/well) onto gelatin coated 48-well plates. For in vitro tests, after ApoF stimulation 50 µL of secretome was pipetted onto gelatin coated plate. After 4 h at 37C, cells were removed by media aspiration, and the wells stained with Coomassie blue. Absorbance for Coomassie blue at 595 nm (SpectraMax M3 Plate Reader) was measured for quantification. The amount of Coomassie blue staining decreased with increased gelatin clearance, and the negative controls were the wells with no PMNs plated. The percentage of tissue clearance was calculated by normalizing absorbance of the negative controls (wells without cells) using the following formula: 1-[(sample Abs/(negative control Abs)].

**Multiplex Cellular Imaging.** For histological analysis of isolated bone marrow PMNs, cells were fixed for 15 min in 4% paraformaldehyde, spun, and reconstituted in ethanol before staining. Blocking was performed in horse serum (Vector Laboratories, S-2000). Cells were incubated in primary antibodies specific for neutrophils (anti-neutrophil mouse monoclonal, Cedarlane CL8993AP; 1:100), NFKBp50 (Millipore 06-886; 1:100), and PPARγ (Abcamab59256; 1:100) each at 4°C for overnight in sequential order, followed by incubation with a horseradish peroxidase (hrp)-conjugated secondary antibodies. After 10 min incubation, opal fluorophores were used to detect fluorescent signal, with the opal 540 used for PMNs, opal 570 for NFKBp50, opal 520 forIRF3, and opal 620 for PPARγ. DAPI was used as a counterstain. Non-specific binding was assessed by performing a no primary antibody control, and cross-over staining was assessed by performing each primary antibody staining on its own section. Images were acquired on the Mantra™ Quantitative Pathology Imaging microscope.

**Clinical Analysis**

**Jackson Heart Study.** The Jackson Heart Study (JHS) consists of 5,306 noninstitutionalized African American participants, 21 years and older, from the Atherosclerosis Risk in the Community (ARIC) site in Jackson, MS. JHS is a representative sample of urban and rural Jackson, MS, metropolitan tri‐county (Hinds, Madison, and Rankin counties) residents, volunteers, randomly selected individuals, and secondary family members of enrolled participants.[12] The current analyses were restricted to 60 participants who were diagnosed with myocardial infarction (MI) before examination 2 (between 2005-2008). Heart failure (HF) events were formally adjudicated by the JHS from January 1, 2005 to December 31, 2012. We have therefore enrolled only those that have been formally adjudicated with HF. Participants who did not have an MI before examination 2, had HF at or before visit 2, were diagnosed with MI after 2008 or did not have plasma collected at examination 2 were excluded, leaving a final sample size of 60 participants. Time between visit 1 and 2 was 5±1 years for both groups.

Participants were divided into 2 groups: 1) those who did not develop HF by 2013 (MI), and 2) those who were diagnosed after 2009 with HF (MI+HF). Classification of HF was based on the following parameters: 1) Framingham criteria equal “heart failure present”, and NHANES criteria equals “heart failure present”, and Modified Boston criteria equal “definite or possible heart failure” and 2) heart failure MMCC classification is either “chronic stable heart failure” or “no heart failure”. For all cohort HF events, review by two physicians was required. If the diagnoses of the two reviewers agreed on diagnosis, the final classification for the occurrence was recorded. If the diagnoses of the two reviews disagreed, a third review by an adjudicator was completed and the adjudicator’s diagnosis was the final classification for occurrence. The JHS was approved by the institutional review boards of the University of Mississippi Medical Center, Jackson State University, and Tugaloo College. All participants provided written informed consent. This protocol was approved as IRB #2014-0274.

**Electrocardiogram Analysis.** Standard 12-lead electrocardiogram (ECG) measurements were recorded at visit 1 and visit 3. The averaged QT interval was then corrected for heart rate using the Bazett formula (QTc = QT/√RR).

**Plasma Glycoproteomics.** Plasma proteins were reduced, alkylated, and trypsin-digested into peptides.[1, 6, 10] The glycopeptides were oxidized and conjugated to a solid support using hydrazide chemistry, and glycopeptides that were previously N-linked were released by PNGase F. Peptides were cleaned using Sep-Pak Vac C18 cartridge (Waters, Milford, MA) and analyzed label-free by liquid chromatography-tandem mass spectrometry using a Q Exactive (ThermoFisher, Waltham, MA) coupled with a 15 cm × 75 μm C18 column (5 μm particles with 100 Å pore size). Samples were run in duplicated and combined for analysis. Target values in MS were 1e6 ions at a resolution setting of 70,000 and in MS2 1e5 ions at a resolution setting of 17,500.

MS/MS spectra were searched with SEQUEST using Proteome Discoverer (version 1.4; Thermo Fisher) against the human RefSeq database (November 3, 2013) containing 53,918 sequences. The false discovery rate (FDR) was set at 0.01 to eliminate low-probability protein identifications. Peptides were quantified using spectral counting and total spectral counts of each sample were used for normalization. Missing values were replaced with a small value (0.01) in order to calculate the ratio and p-value.

**IL6 and IL6R ELISA.**IL6 and IL6R plasma concentration was measured using human IL6 (Novus; NBP2­31051) and IL6R (Novus; NBP1­89870) ELISA kits according to manufacturer instructions.

**Bioinformatics and statistical analysis.**

**mHART bioinformatics analysis**. Plasma and gene data from the mHART database was normalized to respective D0 samples to generate time course and age comparison datasets for functional analysis using Ingenuity Pathway Analysis (IPA; QIAGEN Redwood City; [www.qiagen.com/ingenuity](http://www.qiagen.com/ingenuity)). Heat maps and volcano plots were constructed using a statistical program available in the Metaboanalyst 3.0 package ([www.metaboanalyst.ca/](http://www.metaboanalyst.ca/)).[11, 13] The top features ranked by t-tests were retained to provide the most contrasting patterns.

**Clinical bioinformatics analysis**. For the human glycoproteomic data, the analysis of this study compared sex differences; a separate study using this same sample set was performed to focus on MI and MI+HF differences, where ApoF strongly associated with increased risk for HF (OR 21.84, 95% CI 3.20–149.14).[2] Two group comparisons were analyzed by Students t-test. Statistical significance was set at p<0.05. Partial least squares discriminate analysis (PLSDA) and canonical analysis using IPA was used to visualize sex differences in signaling pathways (supplemental methods). Multiple-testing corrected p values were calculated in IPA using the Benjamini-Hochberg method.

**Supplemental Figures**

**
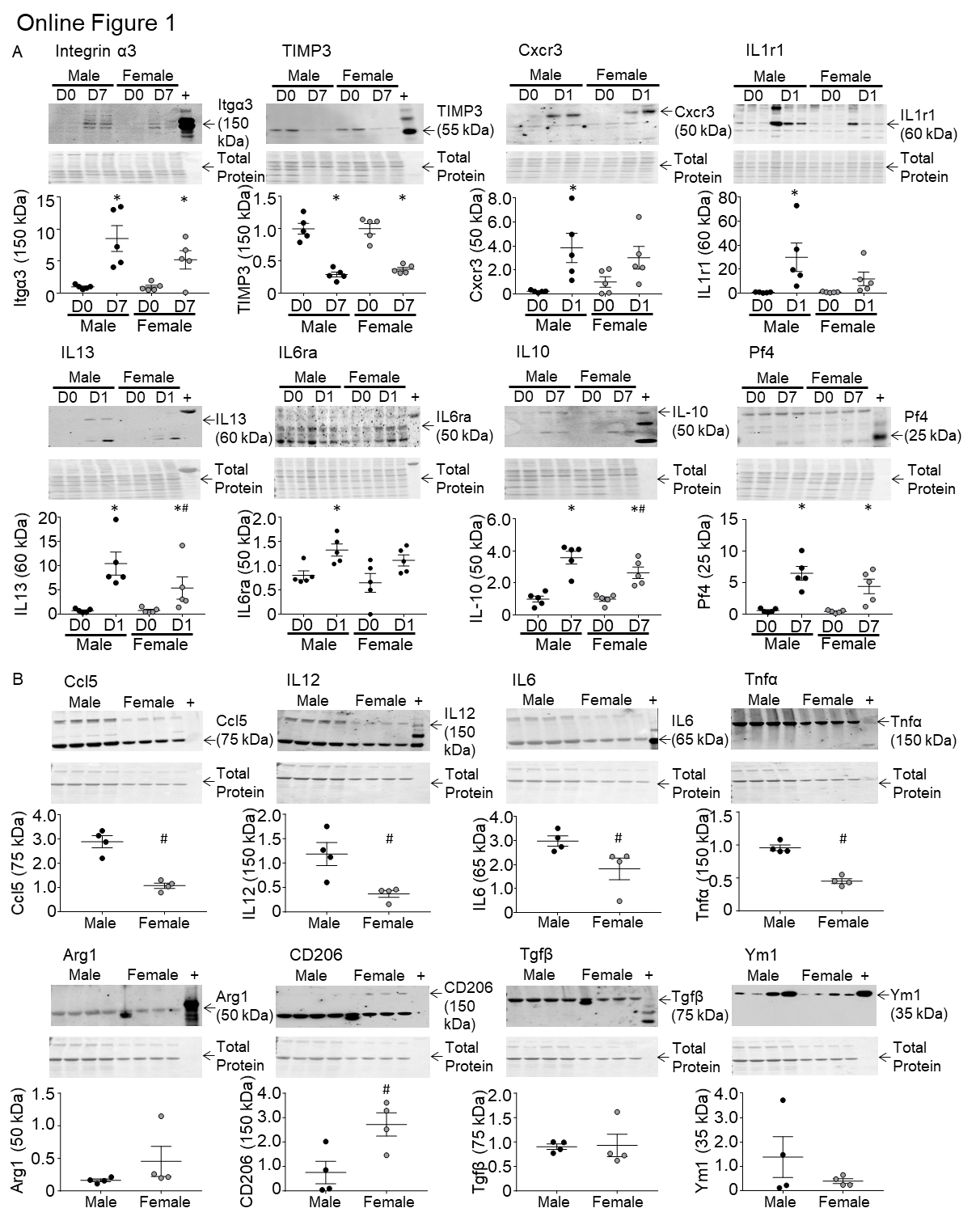
**

**Online Figure 1. LV infarct and neutrophil polarization protein assessment.** (A) A total of 8 proteins were measured by immunoblotting. Integrin α3, TIMP-3, Cxcr3, IL1r1, IL13, and IL6ra were chosen as they showed biggest difference between male and female mice post-myocardial infarction; n=5/group. IL10 and Pf4 were chosen as negative controls since they were not significantly different at the gene level. (B) Immunoblotting of pro-inflammatory (N1) and anti-inflammatory (N2) makers from isolated neutrophils from day 1 infarcts in males and females; n=4/sex; *p<0.05 vs.D0 of respective sex; #p<0.05 vs males. IL- interleukin; LV= left ventricle; Pf4= platelet factor 4; TIMP= tissue inhibitor of metalloproteinase.


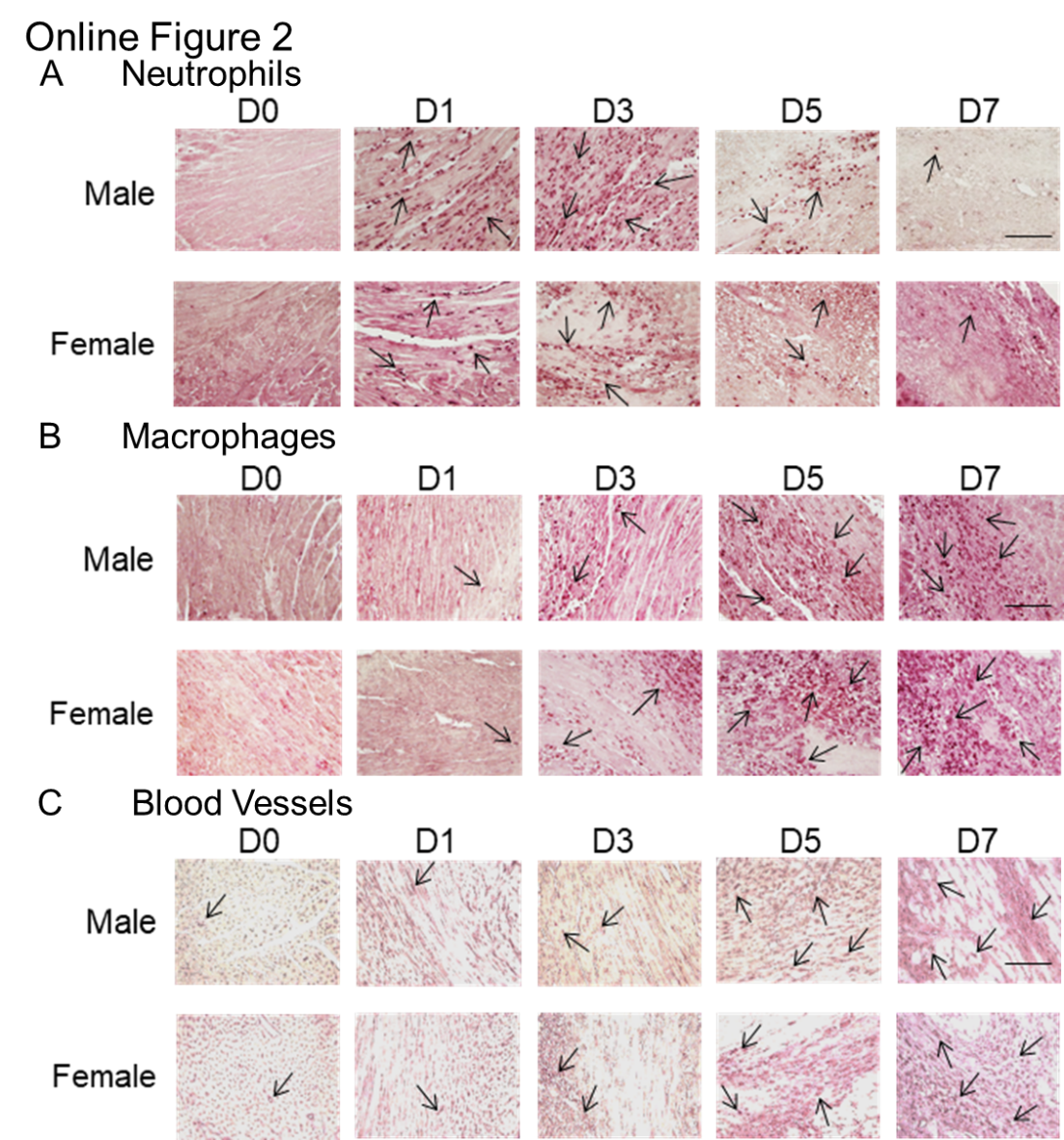


**Online Figure 2. Immunohistochemistry staining of neutrophils, macrophages, and vessel numbers from the mHART database.** (A) Immunohistochemistry within mHART showed neutrophil numbers in the day 3 infarct were lower in female compared to male mice, n≥10/sex/day. (B) No difference was observed in macrophage numbers, n≥10/sex/day. (C) Lectin staining showed slower acceleration of vessel formation in female mice, n≥10/sex/day; Scale bar=60 μm.

**Supplemental Tables**

**Online Table 1.** mHART project data summary. Values represent numbers of individual data sets that were analyzed. Data was downloaded from the mHART database on April 15, 2017 and include all C57BL/6J wild type mice who were either untreated or saline-treated (n=759 total) at days 0, 1, 3, 5, and 7 post-myocardial infarction.

| Echocardiogram | Plasma | Histology | Gene array |
| --- | --- | --- | --- |
| 759 | 282 | 140 | 140 |

**Online Table 2.** Infarct area measurements at days 0, 1, 3, 5, and 7 post-myocardial infarction.

| Day 0 | | Day 1 | | Day 3 | | Day 5 | | Day 7 | | p value |
| --- | --- | --- | --- | --- | --- | --- | --- | --- | --- | --- |
| Male | Female | Male | Female | Male | Female | Male | Female | Male | Female |  |
| N/A | N/A | 57±3% | 60±2% | 57±3% | 53±2% | 59±4% | 55±4% | 59±3% | 55±3% | 0.31 |

**Online Table 3.** Clinical characteristics of men and women at JHS Visit 2 by post-MI congestive heart failure (CHF) status.

| Clinical Characteristic | MI | | CHF | |
| --- | --- | --- | --- | --- |
|  | Men  (n=24) | Women  (n=21) | Men  (n=3) | Women  (n=12) |
| Age, years | 68 ± 8 | 70 ± 9 | 58 ± 9 | 73 ± 8^†^ |
| Incident rate, 100 person-years | NA | NA | 56 ± 34 | 447 ± 292^†^ |
| Time between MI and visit 2 (yrs) | 6 ± 6 | 5 ± 5 | 8 ± 6 | 5 ± 5 |
| Body mass index (kg/m^2^) | 29 ± 4 | 29 ± 5 | 36 ± 2 | 34 ± 11 |
| Smoking (%) | 4 (17%) | 3 (14%) | 1 (33%) | 2 (17%) |
| Kidney disease history (%) | 0 (0%) | 0 (0%) | 1 (33%)* | 1 (8%) |
| Systolic Blood Pressure (mmHg) | 132 ± 21 | 136 ± 15 | 108 ± 8 | 146 ± 24^†^ |
| Diastolic Blood Pressure (mmHg) | 74 ± 11 | 74 ± 7 | 65 ± 7 | 74 ± 11 |
| Diabetes History | 5 (21%) | 5 (24%) | 1 (33%) | 6 (50%) |
| Hemoglobin A1C (%) | 5.9 ± 0.6 | 6.3 ± 0.9 | 9.2 ± 0.9* | 7.3 ± 2.0*^†^ |
| Fasting Glucose Level (mg/dL) | 102 ± 17 | 115 ± 64 | 170 ± 62 | 95 ± 13 |
| Fasting LDL levels (mg/dL) | 89 ± 28 | 117 ± 52 | 91 ± 15 | 104 ± 45 |
| Fasting HDL levels (mg/dL) | 50 ± 19 | 53 ± 11 | 34 ± 6 | 73 ± 37 |
| Statin Use (%) | 11 (46%) | 7 (33%) | 1 (33%) | 6 (50%) |

Mean±SD; *p<0.05 vs. MI of respective sex; ^†^p<0.05 vs Men with MI+HF.

**Online Table 4.** Glycoproteomics Results. Attached Excel file provides intensity unit values for individual samples.

**Online Table 5.** There was strong overlap between gene and protein levels. Arrows show direction of female levels compared to males. A total of 11 out of 16 (69%) were in agreement.

| Target name | Source | Protein | Gene |
| --- | --- | --- | --- |
| Integrin α3 | LVI | ↔ | ↓ |
| TIMP-3 | LVI | ↔ | ↓ |
| Cxcr3 | LVI | ↓ | ↓ |
| IL1r1 | LVI | ↓ | ↓ |
| IL13 | LVI | ↓ | ↓ |
| IL6ra | LVI | ↓ | ↓ |
| IL10 | LVI | ↓ | ↔ |
| Pf4 | LVI | ↔ | ↔ |
| Ccl5 | PMN | ↓ | ↓ |
| IL12 | PMN | ↓ | ↓ |
| IL6 | PMN | ↓ | ↓ |
| Tnfα | PMN | ↓ | ↓ |
| Arg1 | PMN | ↔ | ↑ |
| CD206 | PMN | ↑ | ↑ |
| Tgfβ | PMN | ↔ | ↔ |
| Ym1 | PMN | ↔ | ↓ |

**Online Table 6.** There was strong overlap between pathways and proteins activated post-myocardial infarction in the mouse and community-based cohort data. Arrows show direction of female/women levels compared to males/men. A total of 7 out of 8 (88%) were in agreement.

| Target name | Mouse | Human |
| --- | --- | --- |
| *Pathway* |  |  |
| LXR/RXR Activation | ↓ | ↓ |
| Acute Phase Response Signaling | ↑ | ↓ |
| Production of NO and ROS in Macrophages | ↑ | ↑ |
| IL6 signaling | ↑ | ↑ |
| IL12 signaling | ↔ | ↔ |
| FXR/RXR Activation | ↔ | ↔ |
| *Protein* |  |  |
| Apo C4 | ↓ | ↓ |
| Apo F | ↔ | ↔ |

LXR/RXR= liver X receptor/retinoid X receptor; IL= interleukin; NO= nitric oxide; ROS= reactive oxygenase species; Apo= apolipoprotein

**Supplemental References**

1. DeCoux A, Tian Y, DeLeon-Pennell KY, Nguyen NT, de Castro Bras LE, Flynn ER, Cannon PL, Griswold ME, Jin YF, Puskarich MA, Jones AE, Lindsey ML (2015) Plasma Glycoproteomics Reveals Sepsis Outcomes Linked to Distinct Proteins in Common Pathways. Critical care medicine 43:2049-2058 doi:10.1097/ccm.0000000000001134

2. DeLeon-Pennell K, Ero O, Ma Y, Iyer RP, Flynn E, Espinoza I, Musani S, Vasan R, Hall ME, Fox E, Lindsey ML (2018) Glycosylated Apolipoprotein F predicts development of heart failure following myocardial infarction. J Proteome Res Submitted

3. Deleon-Pennell KY, Bras LE, Lindsey ML (2013) Circulating lipopolysaccharide resets cardiac homeostasis in mice through a matrix metalloproteinase-9 dependent mechanism. Physiol Rep 1:e00079 doi:10.1002/phy2.79

4. DeLeon-Pennell KY, de Castro Bras LE, Iyer RP, Bratton DR, Jin YF, Ripplinger CM, Lindsey ML (2014) P. gingivalis lipopolysaccharide intensifies inflammation post-myocardial infarction through matrix metalloproteinase-9. J Mol Cell Cardiol 76C:218-226 doi:10.1016/j.yjmcc.2014.09.007

5. DeLeon-Pennell KY, Iyer RP, Ma Y, Yabluchanskiy A, Zamilpa R, Chiao YA, Cannon P, Cates C, Flynn ER, Halade GV, de Castro Bras LE, Lindsey ML (2018) The Mouse Heart Attack Research Tool (mHART) 1.0 Database. Am J Physiol Heart Circ Physiol doi:10.1152/ajpheart.00172.2018

6. DeLeon-Pennell KY, Tian Y, Zhang B, Cates CA, Iyer RP, Cannon P, Shah P, Aiyetan P, Halade GV, Ma Y, Flynn E, Zhang Z, Jin YF, Zhang H, Lindsey ML (2016) CD36 Is a Matrix Metalloproteinase-9 Substrate That Stimulates Neutrophil Apoptosis and Removal During Cardiac Remodeling. Circulation. Cardiovascular genetics 9:14-25 doi:10.1161/CIRCGENETICS.115.001249

7. Iyer RP, Patterson NL, Zouein FA, Ma Y, Dive V, de Castro Bras LE, Lindsey ML (2015) Early matrix metalloproteinase-12 inhibition worsens post-myocardial infarction cardiac dysfunction by delaying inflammation resolution. International journal of cardiology 185:198-208 doi:10.1016/j.ijcard.2015.03.054

8. Lindsey ML, Iyer RP, Zamilpa R, Yabluchanskiy A, DeLeon-Pennell KY, Hall ME, Kaplan A, Zouein FA, Bratton D, Flynn ER, Cannon PL, Tian Y, Jin YF, Lange RA, Tokmina-Roszyk D, Fields GB, de Castro Bras LE (2015) A Novel Collagen Matricryptin Reduces Left Ventricular Dilation Post-Myocardial Infarction by Promoting Scar Formation and Angiogenesis. J Am Coll Cardiol 66:1364-1374 doi:10.1016/j.jacc.2015.07.035

9. Ma Y, Yabluchanskiy A, Iyer RP, Cannon PL, Flynn ER, Jung M, Henry J, Cates CA, Deleon-Pennell KY, Lindsey ML (2016) Temporal neutrophil polarization following myocardial infarction. Cardiovasc Res 110:51-61 doi:10.1093/cvr/cvw024

10. Tian Y, Koganti T, Yao Z, Cannon P, Shah P, Pietrovito L, Modesti A, Aiyetan P, DeLeon-Pennell K, Ma Y, Halade GV, Hicks C, Zhang H, Lindsey ML (2014) Cardiac extracellular proteome profiling and membrane topology analysis using glycoproteomics. Proteomics Clin Appl 8:595-602 doi:10.1002/prca.201400009

11. Vu TH, Shipley JM, Bergers G, Berger JE, Helms JA, Hanahan D, Shapiro SD, Senior RM, Werb Z (1998) MMP-9/gelatinase B is a key regulator of growth plate angiogenesis and apoptosis of hypertrophic chondrocytes. Cell 93:411-422 doi:org/10.1016/S0092-8674(00)81169-1

12. Wilson JG, Rotimi CN, Ekunwe L, Royal CD, Crump ME, Wyatt SB, Steffes MW, Adeyemo A, Zhou J, Taylor HA, Jr., Jaquish C (2005) Study design for genetic analysis in the Jackson Heart Study. Ethn Dis 15:S6-30-37

13. Xia J, Sinelnikov IV, Han B, Wishart DS (2015) MetaboAnalyst 3.0--making metabolomics more meaningful. Nucleic Acids Res 43:W251-257 doi:10.1093/nar/gkv380

14. Zamilpa R, Zhang J, Chiao YA, de Castro Bras LE, Halade GV, Ma Y, Hacker SO, Lindsey ML (2013) Cardiac wound healing post-myocardial infarction: a novel method to target extracellular matrix remodeling in the left ventricle. Methods in molecular biology 1037:313-324 doi:10.1007/978-1-62703-505-7_18
